# Occupational differences in the prevalence and severity of long-COVID: analysis of the Coronavirus (COVID-19) Infection Survey

**DOI:** 10.1136/oemed-2023-108930

**Published:** 2023-09-28

**Authors:** Theocharis Kromydas, Evangelia Demou, Rhiannon Edge, Matthew Gittins, Srinivasa Vittal Katikireddi, Neil Pearce, Martie van Tongeren, Jack Wilkinson, Sarah Rhodes

**Affiliations:** 1 MRC/CSO Social and Public Health Sciences Unit, School of Health and Wellbeing, University of Glasgow, Glasgow, UK; 2 Lancaster Medical School, Lancaster University, Lancaster, UK; 3 Centre for Biostatistics, The University of Manchester, Manchester, UK; 4 Faculty of Public Health, London School of Hygiene & Tropical Medicine, London, UK; 5 Department of Medical Statistics, London School of Hygiene & Tropical Medicine, London, UK; 6 Centre for Occupational and Environmental Health, The University of Manchester, Manchester, UK; 7 Thomas Ashton Institute for Risk and Regulatory Research, The University of Manchester, Manchester, UK

**Keywords:** COVID-19, Epidemiology, Occupational Health, Longitudinal studies, Materials, exposures or occupational groups

## Abstract

**Objectives:**

To establish whether prevalence and severity of long-COVID symptoms vary by industry and occupation.

**Methods:**

We used Office for National Statistics COVID-19 Infection Survey (CIS) data (February 2021–April 2022) of working-age participants (16–65 years). Exposures were industry, occupation and major Standard Occupational Classification (SOC) group. Outcomes were self-reported: (1) long-COVID symptoms and (2) reduced function due to long-COVID. Binary (outcome 1) and ordered (outcome 2) logistic regression were used to estimate odds ratios (OR)and prevalence (marginal means).

**Results:**

Public facing industries, including teaching and education, social care, healthcare, civil service, retail and transport industries and occupations, had the highest likelihood of long-COVID. By major SOC group, those in caring, leisure and other services (OR 1.44, 95% CIs 1.38 to 1.52) had substantially elevated odds than average. For almost all exposures, the pattern of ORs for long-COVID symptoms followed SARS-CoV-2 infections, except for professional occupations (eg, some healthcare, education, scientific occupations) (infection: OR<1; long-COVID: OR>1). The probability of reporting long-COVID for industry ranged from 7.7% (financial services) to 11.6% (teaching and education); whereas the prevalence of reduced function by ‘a lot’ ranged from 17.1% (arts, entertainment and recreation) to 22%–23% (teaching and education and armed forces) and to 27% (not working).

**Conclusions:**

The risk and prevalence of long-COVID differs across industries and occupations. Generally, it appears that likelihood of developing long-COVID symptoms follows likelihood of SARS-CoV-2 infection, except for professional occupations. These findings highlight sectors and occupations where further research is needed to understand the occupational factors resulting in long-COVID.

**WHAT IS ALREADY KNOWN ON THIS TOPIC**

- SARS-CoV-2 infection and COVID-19 mortality in the UK varied by occupational group; yet it is not known if any occupational groups are more susceptible to long-COVID than others.

**WHAT THIS STUDY ADDS**

- This is the first study to examine how prevalence of long-COVID and its impacts on functional capacity differ by industry and occupational groups.
- Prevalence of self-reported long-COVID increased with time across all exposure groups and mostly followed SARS-CoV-2 infection trends; with the exception of professional occupations (eg, healthcare, education, scientific occupations) that demonstrated notable differences in the direction of odds of long-covid when compared with odds of SARS-CoV-2 infection.
- Those working in teaching and education, and social care industries showed the highest likelihood of having long-COVID symptoms. The exact same pattern was observed when analysis was performed using occupational groups. When we used Standard Occupational Classification groups, the likelihood was higher in Caring, leisure and other services.

**HOW THIS STUDY MIGHT AFFECT RESEARCH, PRACTICE OR POLICY**

- The findings contribute to the evidence base that long-COVID differences occur across industries and occupations, provides insights for employees, employers, occupational and healthcare for the industries and occupations that may need additional support for return-to-work policies and highlights sectors and occupations where further research is needed to understand the mechanisms resulting in long-COVID, how occupational factors influence the risk of developing long-COVID or interact with long-COVID to increase the impact on activities and where further protective measures are needed.

## Introduction

In the UK, the risk of SARS-CoV-2 infection and COVID-19 mortality has varied by occupational group, although differences appear to have declined over the duration of the pandemic.^1–5^ It remains unclear; however, whether or not some occupational groups are more susceptible to long-COVID; and if any differences reflect, or are addition to, differential risks in SARS-CoV-2 infection.^6^ The highest risks of long-COVID are reported among workers in education, social care and healthcare sectors^7^; all sectors with elevated risks of SARS-CoV-2 infection during the pandemic.^8^

Being out of work is associated with poor health^9–11^ and the risk of unemployment increases with length of sick leave.^12^ Recent studies have demonstrated the burden of long-COVID and its impact on return to previous levels of work.^13 14^ A Danish nationwide registry study demonstrated that of those hospitalised, being female, older age and having a comorbidity were associated with a lower chance of returning to work.^15^ Davis *et al* showed that almost half of the study respondents suffering with long-COVID required a reduced work schedule compared with preillness.^13^ A Swedish national cohort study showed that 13% were on sick leave due to long-COVID from March to August 2020 and 9% were on sick leave for at least 4 months.^16^ Those on sick leave due to long-COVID were older, predominantly men, had higher sick leave prior to COVID-19 and were more likely to have received inpatient care.^16^ While we understand some of the factors that predict long-COVID and work ability, little is known about how the prevalence of long-COVID and associated functional capacity differs by occupation and the impact of occupational factors on these patterns.

Long-COVID has a disproportionate impact on groups already disadvantaged in terms of work and health. Returning to work is part of rehabilitation from illness and is important to those recovering from long-COVID.^12^ Those with significant illness or disability can work effectively if provided with suitable support.^12^ Better understanding of the prevalence and severity of long-COVID across occupations will inform the need for rehabilitation and workplace adjustment measures^17^ and whether or not long-COVID should be considered an occupational disease.^7^ It may also identify workplaces that need additional support measures to reduce risks of transitioning to long-COVID after SARS-CoV-2 infection.

Our study aims to establish whether the prevalence and severity of long-COVID varied by occupation and if any differences are in excess of differences in the risk of SARS-CoV-2 infection.

## Methods and data

### Study design

We used data from the Office for National Statistics (ONS) COVID-19 Infection Survey (CIS) from February 2021 (long-Covid questions first included in CIS) to the end of April 2022. The CIS began in April 2020, used random sampling and aimed to be representative of the UK population. Repeated PCR testing for all participants was carried out during weekly home visits for the first month of entry to the survey, and then monthly until 30 April 2022 when testing samples were sent and returned by post and survey questions answered on-line or by phone.^18^ CIS uses four different variables to capture employment status. Participants were coded as employed or not working (online supplemental file 1 'Employment status coding' section).

10.1136/oemed-2023-108930.supp1 Supplementary data

Analyses were restricted to participants aged ≥16 to ≤65 years old on the day of their first home visit and to those who turned 16 during the study period and had answered long-COVID questions at least once.

We used the first available observation per occupational exposure and other covariates, assuming no change across time. In sensitivity analyses, we estimated panel models to allow for time variation.

### Exposure groups

We used three different industry and occupational groupings. Industry was classified based on the ONS sector groupings used within CIS.^19^ Based on expert judgement and consensus among team members, we derived a bespoke set of occupational groupings using four-digit Standard Occupational Classification (SOC 2010) (online supplemental tables S1 and S2).^20^ Groups used in previous studies^2–4 21^ focused on key worker/essential workers (ie, those with highest exposure at the start of the pandemic), and therefore, new groupings were needed to capture all occupations and exposures while ensuring adequate group size, as the pandemic progressed. The third exposure category was by major SOC group.

### Analytical sample and ascertainment of outcome

Primary outcome measures were self-reported (1) cases of long-COVID and (2) reduced function (ie, severity) due to long-COVID among those reporting long-COVID, using the following CIS questions:

1. Self-reported long-COVID: ‘Would you describe yourself as having ‘long-COVID’, that is, you are still experiencing symptoms more than 4 weeks after you first had COVID-19, that are not explained by something else?’. For our primary analysis, we reported the n (%) of people who self-reported having long-COVID in at least one survey, irrespective of any COVID-19 test result or previous infection reported.
2. Self-reported reduced function (severity) of long-COVID: ‘How much did long-COVID reduce your ability to carry out daily activities?’ Respondents could answer 0: not at all, 1: yes, a little, 2: yes, a lot. This analytical sample was restricted to those self-reporting long-covid symptoms.

### Statistical analyses

Analysis was completed for the two outcome variables using all three exposure groups, irrespective of whether they have tested or reported being previously infected by SARS-CoV-2. For comparison, we also performed analyses using the outcome ‘SARS-CoV-2 infection (self-reported or positive PCR test).

Sample characteristics were summarised using frequencies and proportions. Descriptive statistics were used to show the change in prevalence of self-reported long-COVID over time. Binary (outcome 1: long-COVID symptoms) and ordered (outcome 2: reduced function due to long-COVID) logistic regression models with robust standard errors to account for heteroskedasticity were used. For the binary logistic regression analyses, data were collapsed by person using the first reported values for exposure and covariates and taking ‘at least one reported episode of long-COVID’ as the outcome. For the ordered logistic regression, the highest reported value for the outcome variable (ie, severity of long-COVID) was used per person. We used effects coding where ORs are contrasted to each group mean compared with the grand mean showing the likelihood of having long-COVID in each group. To assess for potential confounding or mediating differences, we estimated unadjusted and fully adjusted models for all the covariates (age, sex, index of multiple deprivation, rural or urban location, household size, region, pre-existing health conditions).^2^

**Figure 1:**
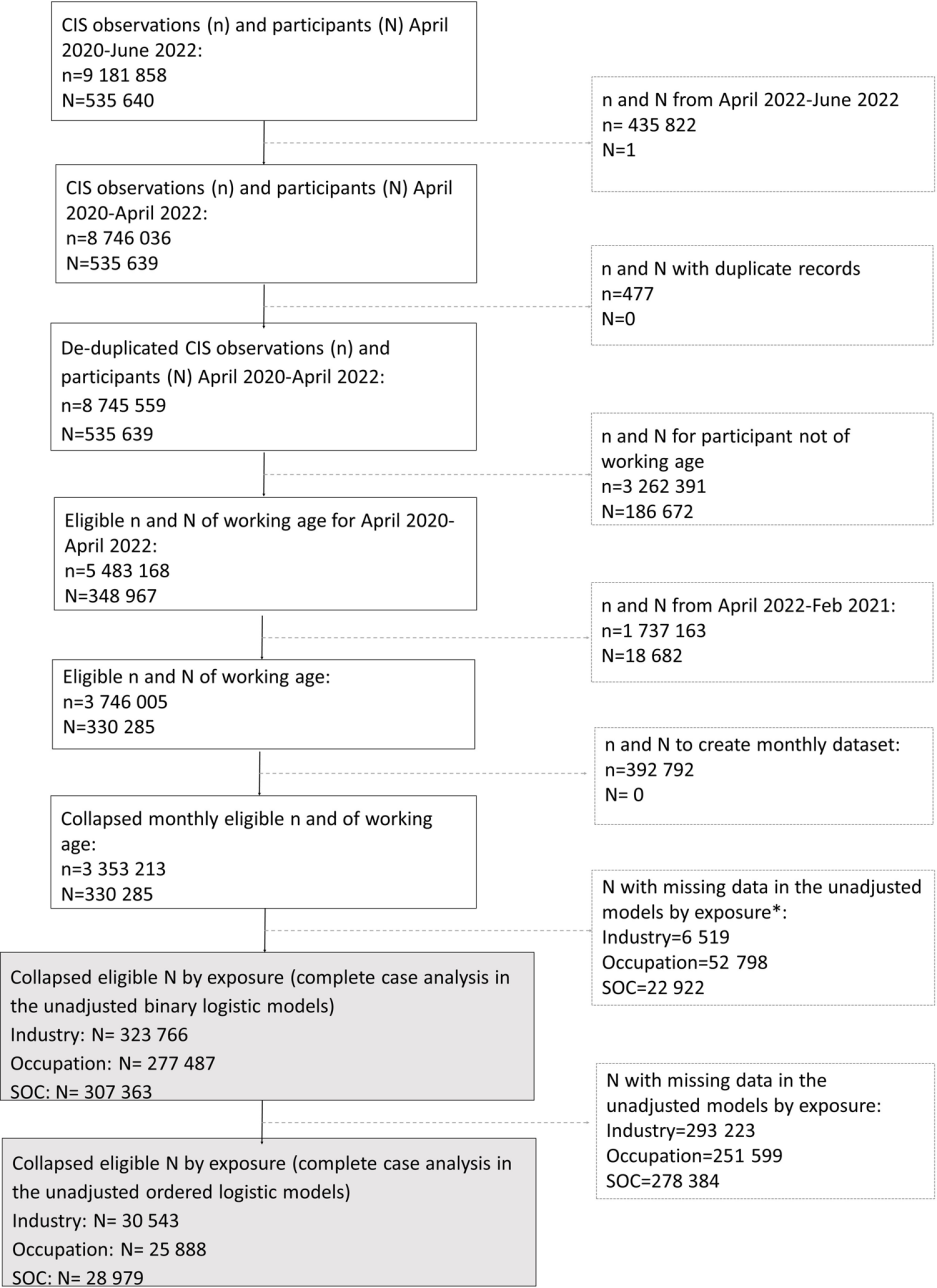
Flow chart of COVID-19 Infection Survey (CIS) participants. N=number of participants, n=number of observations. *Data missingness higher for occupation (four-digit SOC) and major SOC groups than for industry. SOC, Standard Occupational Classification.

Weighting was not used as the CIS derived weights are cross-sectional. Testing for patterns of missingness in the outcome variable did not show any differences (online supplemental tables S3 and S4). Participants with no measures in our outcome, exposure and covariate variables were excluded (figure 1; online supplemental tables S3 and S4). We included the non-working group for comparison purposes.

### Sensitivity analyses

Sensitivity analyses were conducted using two additional analytical samples: (1) those with a positive PCR test and (2) those with a positive PCR test and/or self-reported infection before entering the CIS. Furthermore, using the specifications of our main sample, a panel dataset was created where multiple participant visits and records were merged to create a dataset with one observation per month per person to allow for panel analysis that accounts for variation across time for time-varying variables, for example, ccupation, employment status (figure 1). We used multilevel mixed-methods generalised linear models, with a binomial link function for the outcome of having long-COVID symptoms; and a multilevel mixed-effects ordered logistic regression for the outcome of reduced function due to long-COVID and compared with the estimations of our analytical model.

All analyses were calculated by using STATA V.17.^22^

## Results

### Cohort characteristics

Our final analytical samples included 323 766 individuals by industry, 277 487 individuals by occupational group and 307 363 individuals by major SOC group (figure 1). Online supplemental table S2 illustrates descriptive statistics by exposure group. In all cases, the non-working group had approximately twice as high prevalence of underlying health conditions (online supplemental table S5) and a higher prevalence of being affected ‘a lot’ by long-COVID (online supplemental tables S6–S8) compared with the working group.

**Figure 2:**
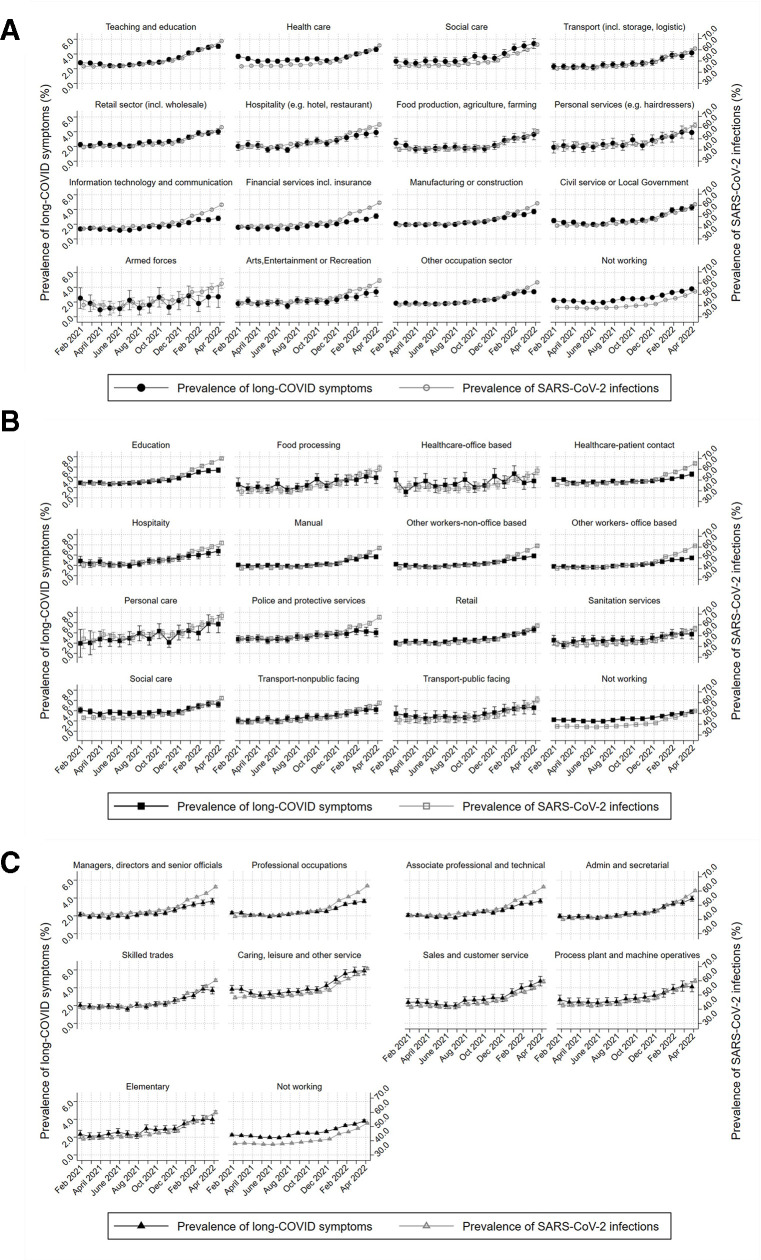
Monthly prevalence (%) of self-reported long-COVID symptoms (primary y-axis; left) and SARS-CoV-2 infections (secondary y-axis; right) for all workers aged 16–65 for (A) industry; (B) occupational groups and (C) SOC major groups. SOC, Standard Occupational Classification.

Monthly prevalence of self-reported long-COVID and SARS-CoV-2 infection across time and across all three occupational exposures, demonstrates an increase of the prevalence of self-reported long-COVID with time, following similar trends in SARS-CoV-2 infection (figure 2). Prevalence of long-COVID by industry ranges from ~2% to almost 6% in April 2022 for social care and education. The healthcare sector also shows relatively high prevalence that increases post-January 2022. Similar patterns in long-COVID prevalence are observed by occupation and major SOC group. Education and social care and hospitality occupations demonstrate the highest prevalence (>4% from January 2022). Caring, leisure and other service occupations (SOC group) exhibit the highest prevalence, followed by sales and customer service occupations.

### Likelihood (ORs) and prevalence (marginal means) of long-COVID

**Figure 3:**
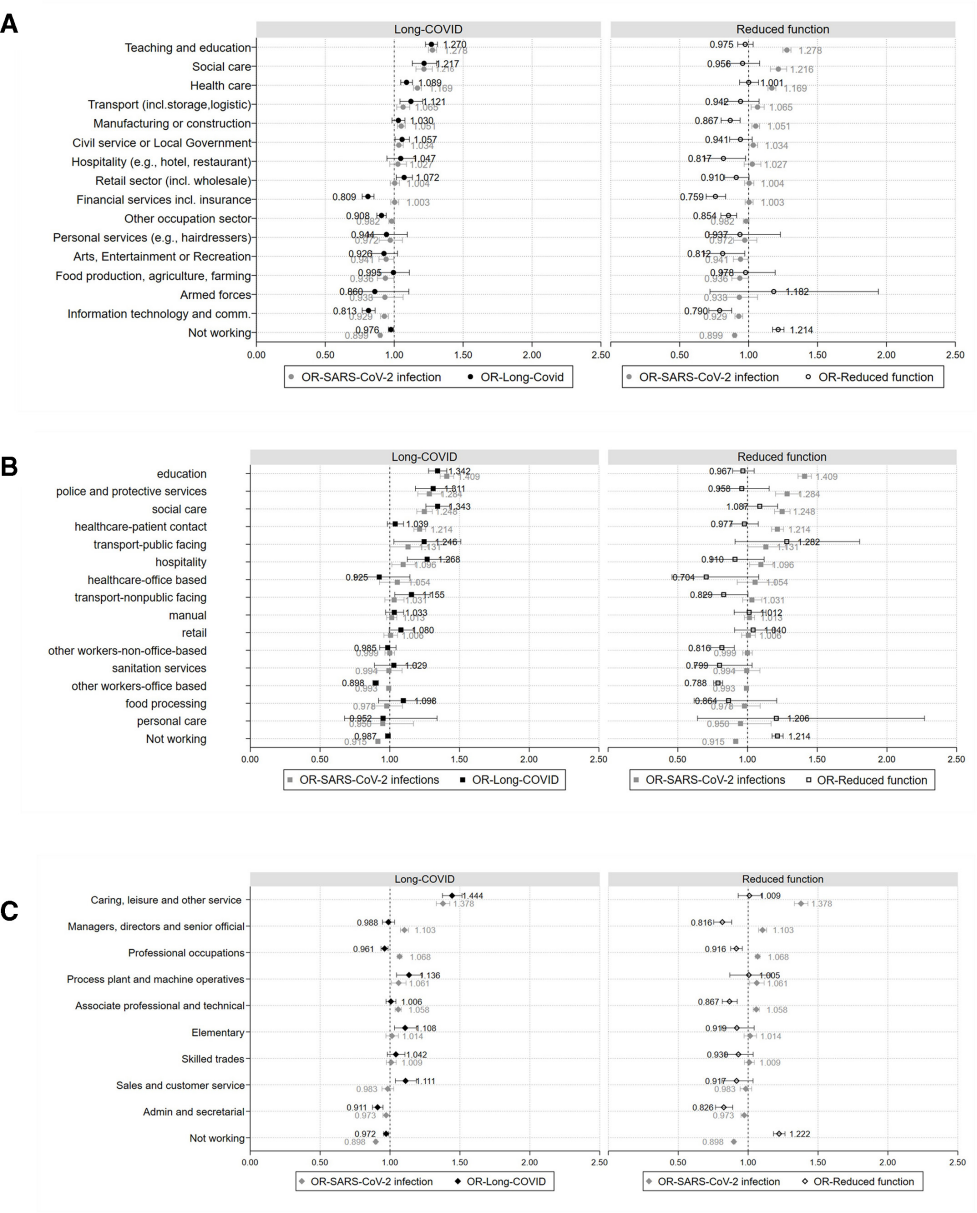
Odds (A–C) of having Long-COVID symptoms (left) and reduced function (right) by industry, occupational groupings and major SOC groups. SOC, Standard Occupational Classification.

In the unadjusted model (online supplemental tables S9 and S10), teaching and education (OR 1.41; 95% CI: 1.36 to 1.46), social care (1.38; 95% CI 1.28 to 1.48), healthcare (1.19; 95% CI 1.14 to 1.23), civil service or local government (1.12; 95% CI 1.06 to 1.17), retail (1.08; 95% CI 1.03 to 1.14) and transport (1.08; 95% CI 1.00 to 1.16) demonstrated increased likelihood of reporting long-COVID symptoms compared with the average working-age population. All other sectors (including the non-working group) showed ORs less than one or with very wide CIs, with the lowest ORs being in the information technology (IT) and communication sector (0.75; 95% CI 0.71 to 0.79). In the fully adjusted model (online supplemental tables S9 and S10, figure 3A), ORs for most industries were attenuated and the pattern remained the same with teaching and education exhibiting the highest odds (1.27; 95% CI 1.23 to 1.31). Exceptions to confounding attenuation were primarily seen for the transport industry (1.12; 95% CI 1.04 to 1.21) and hospitality and manufacturing, although with wide CIs. Prevalence of long-COVID (marginal means-predicted probability of having at least one episode of long-COVID symptoms across all groups) ranged from 7.8% (IT, financial services and armed forces) to 11.1% (social care) and 11.6% for teaching and education (online supplemental table S11 and figure S1A).

By occupational group, in the unadjusted model most demonstrated higher likelihood of having long-COVID; however, for some CIs are wide (online supplemental table S9). Similar to analysis by industry, education (1.53; 95% CI 1.61 to 1.79) and social care (1.53; 95% CI 1.45 to 1.60) occupations showed the highest risk. Manual, other workers-non-office based and office-based occupations, along with the non-working group showed ORs<1. After adjustment (online supplemental table S9, figure 3B), education (1.34; 95% CI 1.28 to 1.41) and social care (1.34; 95% CI 1.26 to 1.43) remained the two groups with the highest ORs and were closely followed by police and protective services (1.31; 95% CI 1.18 to 1.45), hospitality (1.27; 95% CI 1.13 to 1.43) and transport public facing (1.25; 95% CI 1.03 to 1.51) or non-public facing (1.16; 95% CI 1.04 to 1.29). The group with the lowest ORs was office-based occupations (0.90; 95% CI 0.88 to 0.92). All other groups with ORs≤1 have very wide confidence intervals. Prevalence of long-COVID by occupational group ranged from a low of 8.4% in other workers-office based, to a high of 12% in education (online supplemental table S11 and figure S1B).

Using major SOC group as the exposure, in the unadjusted model (online supplemental table S9), ORs for Caring, Leisure and other services stand out (1.65; 95% CI 1.58 to 1.73), compared with all other groups, followed by the sales and customers service, elementary and process plant and machine operatives’ groups with ORs>1. In the remaining major SOC groups, ORs were lower than one. After adjustment the category with the highest ORs remains the same; however, ORs were slightly attenuated. Minor changes were observed in all other groups (online supplemental table S9, figure 3C). Admin and secretarial, professional occupations (eg, healthcare, education, scientific occupations) and those not working were less likely to report long-COVID symptoms. However, those in caring, leisure and other services (1.44, 95% CI 1.38 to 1.53) still demonstrated substantially higher odds than average, followed by process plant and machine operatives (1.14, 95% CI 1.05 to 1.23), sales and customer service (1.11, 95% CI 1.04 to 1.19) and elementary occupations (1.11, 95% CI 1.04 to 1.19). Finally, estimations for managers, directors and senior officials, and associate professional and technical occupations are uncertain. For most occupational groups prevalence of long-COVID was between 9% and 10%, with the exceptions of caring, leisure and other service occupations with 13% prevalence analysis by major SOC group (online supplemental table S11 and figure S1C).

Comparing the likelihood of long-COVID with the likelihood of SARS-CoV-2 infection (figure 3A–C) demonstrates similar patterns, that is, industries with high risk of SARS-CoV-2 infection generally display high likelihood of long-COVID symptoms. In the majority of occupational groups similar patterns exist, with minor discrepancies for healthcare-office based where OR>1 for SARS-CoV-2 infection and OR<1 for long-COVID; and food processing and sanitation services where the opposite pattern is observed (figure 3B). Using major SOC groups as the exposure displayed similar findings (figure 3C), with the main exception of professional occupations, where likelihood of long-COVID is proportionally higher compared with SARS-CoV-2 infection. Some indications of different patterns between risk of infection and long-COVID were also observed in managers, directors and senior officials and sales occupations; however, in all cases confidence intervals are wide.

### Likelihood (ORs) and prevalence (margins) of reduced function due to long-COVID

In order to examine the extent to which long-COVID symptoms impacted daily activities (ie, severity), we used a reduced sample for all three exposure groups with analyses restricted to those self-reporting at least one long-COVID symptom over the study period. Final analytical samples were 30 543 participants for industry, 25 888 participants for occupational groups and 28 979 participants for major SOC groups (figure 1).

In the unadjusted model (online supplemental table S10) all industries, apart from healthcare (1.07; 95% CI 1.00 to 1.14) and social care (1.07; 95% CI 0.95 to 1.14), demonstrated lower odds of experiencing reduced function (ie, reporting that daily activities were impacted a little or a lot) compared with the grand mean, while those not working had the highest odds (1.26; 95% CI 1.23 to 2.03). Adjusting for all covariates attenuates ORs and in many cases CIs are wide (eg, retail sector, food production and personal services); however, the trends remain stable (online supplemental table S10, figure 3A).

Analysis by occupational groups (online supplemental table S10), similarly demonstrated that those not working have very high odds of reduced function (1.25; 95% CI 1.21 to 1.29); however, the highest odds are observed in the transport-public facing occupational group (1.29; 95% CI 0.92 to 1.81), followed by personal (1.25; 95% CI 0.67 to 2.32) and social care (1.23; 95% CI 1.10– to 1.37) occupations. In all other groups, ORs are less than 1. Adjusting for all confounders generally attenuated the odds, but results retained the same patterns for magnitude and CIs (online supplemental table S10, figure 3A).

By major SOC group, in the unadjusted model (online supplemental table S10) for almost all groups, it is less likely for someone to demonstrate reduced function due to long-COVID. The exceptions were the not working group (1.27; 95% CI 1.23 to 1.31) and the caring, leisure and other services (1.09; 95% CI 1.01 to 1.18). Following adjustment, effect is attenuated for all groups (online supplemental table S10, figure 3A).

Prevalence of reduced function by a ‘a little’ ranged between 47% and 49% for all three exposure groups (online supplemental table S11 and figure S2). Prevalence of reduced function by ‘a lot’, ranged from 17.1% to 21.7% by industry (for the arts, entertainment or recreation and teaching and education sectors, respectively), and 27.3% for those not working. By occupational group, the lowest prevalence of reduced function by ‘a lot’ was observed for education (24.1%) and the highest for social care, personal care and public-facing transport occupations. The latter were comparable to the prevalence for those not working (28.2%) (online supplemental table S11 and figure S2). By major SOC group, reduced function by ‘a lot’ ranged from 20.9% for managers, directors and senior officials and admin and secretarial to 24.4% for caring, leisure and other services and process plant and machine operatives.

Analysis run on the specified two additional samples, that is, (1) confirmed PCR test or (2) confirmed PCR test or prior report of infection (online supplemental figures S3 and S4) did not show different patterns, confirming the robustness of our results.

For long-COVID, ORs from the panel analysis were relatively larger compared with our main analysis for almost all industrial and occupational groups. The opposite was observed for major SOC groups, although the magnitude of the ORs were closer to those in our main sample. Similar patterns were observed for the outcome of reduced daily function (online supplemental tables S12–S14), suggesting assumptions about changes in occupation/occupational status over time are valid.

## Discussion

### Summary of findings

The prevalence of self-reported long-COVID increased through the study period. The social care and education industry (~6%) and hospitality (~4%) occupations recorded the highest prevalence. Industries and occupations with high levels of office-based working (eg, IT), displayed lowest odds of long-COVID compared with more public facing industries, including teaching and education, social care, healthcare, civil service, retail and transport industries and occupations. By major SOC group, those in caring, leisure and other services (1.44; 95% CI 1.38 to 1.52) and education displayed (1.34; 95% CI 1.28 to 1.41) substantially higher odds than average. The likelihood of reporting long-COVID symptoms followed the trend of risk of infection for most industries, occupational groups and SOC groups. The exception was for professional occupations, where the odds of reporting long-COVID symptoms was higher than that of infections. Indications of differing patterns between infection and long-COVID were also observed in other occupational groups, but uncertainty was high. Long-COVID symptoms impacted almost half of all affected participants’ ability to do daily activities by ‘a little’. Participants in healthcare and social care industries were most impacted by ‘a lot’. Similarly, occupational groups most affected were public-facing occupations with those working in transport occupations having the highest prevalence of all (30%), followed by the not working (28.1%) and personal care (27.4%). For SOC groups the highest prevalence (24%) was observed in caring, leisure and other services and process plant and machine operatives.

### Findings in context with previous literature

To our knowledge, no other studies examine long-COVID across industries and/or occupations for direct comparisons. However, previous work estimated that ~10% of people infected with SARS-CoV-2 may have significant postacute or chronic symptoms persisting >12 weeks^23^ and that a substantial proportion will have symptoms that are disabling and involve prolonged absence from work.^24^ These findings are in line with our results.^23 24^ A study in adult hospital patients with COVID-19 showed that postacute COVID-19 syndrome was detected in half of COVID-19 survivors.^25^ These are higher than our results but were an older cohort and probably had severe COVID-19 infection.^25^ Vulnerable groups are disproportionately represented among essential workers (eg, bus drivers, allied health professionals).^26 27^ In addition to being at greater risk of infection^3 4 28 29^ and mortality,^30–32^ they may also face a disproportionate burden of long-COVID. Our findings suggest that workers in social care, education, hospitality, food production and transport have an increased likelihood of long-COVID. Groups likely to have high levels of long-COVID include those with a high past risk of SARS-CoV2 infection^2–4 26 28 30^ and mortality,^30–32^ with the exception of professional occupations.

### Strengths and limitations

Our study has several strengths. We used a large nationally representative dataset to examine prevalence and risk of long-COVID across industries, and occupations, which fills an important knowledge gap. Our analysis included a relatively long study period (February 2021–April 2022), allowing us to examine trends over time. Some limitations should be noted. Any analysis by occupation/industry requires aggregation of different types of workers. It is possible that analyses mask differences within particular sectors or occupations. Opting to use effect coding allows to compare each exposure group to the grand mean. Other methodological approaches may likely produce slightly different results driven by multiple comparison adjustment testing. Furthermore, differences in mitigation and protective measures across countries may lead to different results and comparable nationally studies are needed. In our main analyses, we used occupational status at the beginning of the study period, which may mask effects due to changes in employment status. However, our sensitivity analysis using panel data addressed this. At the time of data collection, there was no universal definition of long-COVID, and the results rely on self-reported measures which are prone to bias. It is possible that bias in this measure is related to occupation or due to other health condition. Furthermore, the discrepancy in duration of symptoms asked in the CIS (ie, >4 weeks) vs the latest WHO definition (ie, symptoms that last for at least 2 months) means that some overestimation of cases may have occurred.^33^ The CIS recording of COVID-19 infections does not include infections occurring between visits and missed visits may be related to occupation, for example, due to shift work. It is not possible to assess the reason(s) participants are not working. Therefore, we cannot examine or exclude the possibility that some participants are not working due to long-COVID.

## Conclusions

The SARS-CoV-2 pandemic created new challenges for workers, employers, occupational and public health. Long-COVID is compounding to these challenges, mainly related to sustaining employment and return-to-work for those affected. Our findings show that long-COVID differences are evident across industries and occupations. Professional occupations, including jobs in teaching and education, IT, welfare and healthcare, demonstrated that the risk of infection may not be the only driving force. Our findings of increased risk of long-COVID in the health and social care industries support that some health conditions are prescribed as industrial diseases^7^ and highlight other sectors and occupations that require further attention. Scientific evidence is essential to understand the mechanisms resulting in long-COVID, how occupation influences prevalence and severity and whether working conditions and protective measures affect the risk of developing long-COVID or interact with long-COVID to increase the impact on activities.

## Data Availability

Data may be obtained from a third party and are not publicly available. ONS CIS data can be accessed only by researchers who are Office of National Statistics (ONS) accredited researchers. Researchers can apply for accreditation through the Research Accreditation Service. Access is through the Secure Research Service (SRS) and approved on a project basis. For further details see: https://www.ons.gov.uk/aboutus/whatwedo/statistics/requestingstatistics/approvedresearcherscheme.
