# Supplementary material for: Occupational differences in the prevalence and severity of long-COVID: analysis of the Coronavirus (COVID-19) Infection Survey
Source: Occup Environ Med. Author manuscript; Available in PMC 2023 Oct 13. (PMC7615205; doi:10.1136/oemed-2023-108930)
Supplement: Supplementary file 1 [file EMS188935-supplement-Supplementary_Material.pdf]

Occupational differences in the prevalence and severity of long-COVID: Analysis of the Coronavirus (COVID-19) Infection Survey

[Supplementary Material]

Employment status coding

The working group included those employed and self-employed XYZ and the not working those who reported being students, furloughed (temporarily not working), unemployed, retired, long-term sick etc, those that are employed or self-employed but currently are not working for reason other than furlough, those looking for paid work being able start and those not working but also not looking for work. First variable used: work_status_clean (0:Not working, 1: Working, 2: Student (**0 and 2 were moved to Not working)**

Second variable used: work_status (1: Employed, 2: Self-employed, 3:Furloughed (temporarily not working), 4: Not working (unemployed, retired, long-term sick etc), 5: Student (**3,4 and 5 were moved to Not working)**

Third variable used: work_status_v1: (1: Employed and currently working, 2: Employed and currently not working, 3: Self-Employed and currently working, 4: Self-Employed and currently not working, 5: Looking for paid work and able to start, 6: Not working and not looking for work, 7: Retired, 8: Child under 5y not attending child care, 9: Child under 5y attending child care, 10: 5y old and older in full-time education (**2,4 and 5 to 10 were moved to Not working)**

Fourth variable used: work_status_v2: (1: Employed and currently working, 2: Employed and currently not working, 3: Self-Employed and currently working, 4: Self-Employed and currently not working, 5: Looking for paid work and able to start, 6: Not working and not looking for work, 7: Retired, 8: Child under 4-5y not attending child care, 9: Child under 4-5y attending child care, 10: 5y old and older at school/home-school, 11: Attending college or FE (including if temporary absent), 12: Attending university (including if temporary absent (**2,4 and 5 to 12 were moved to Not working)**

Handling of duplicates

We identified multiple duplicate observations (947) for individuals on the same date (e.g., for additional blood tests). To address this, first, we replaced Follow up (1) to First visit (0) where visit number equalled zero and visit date was the same (105 observations). Then, seven observations were changed to follow up visit value (1) from First visit (0) as these occurred after the original First visit. Finally, we deleted the remaining duplicates (477 observations (), creating our final dataset with one observation per individual. This analysis allowed for variables' values to change across time.

Occupations were grouped based on a consensus building exercise between members of the research team and other experts that expanded on the essential workers grouping previously used in the Mutambudzi et al study (1) to reflect potential exposures during the initial phase of the pandemic. Using SOC 2010 4 digit codes, the consensus produced eleven main occupational groups which were further split to 15 subgroups (Occ_subgroups; Table S 1). The groupings were also informed by the the COVID-19-JEM *(Oude Hengel KM, et al. Scand J Work Environ Health 2022;48:61-70).*

Table S1 Occupational groupings

**Occupational Group**	**Occupational Sub-group**	**SOC2010 4-digit code**
food processing	food processing	5431, 5432, 5433, 8111, 9272
healthcare	healthcare-office based	1181, 1241, 4211
	healthcare-patient contact	2211, 2212, 2213, 2214, 2215, 2217, 2218, 2219, 2221, 2222, 2223, 2229, 2231, 2232, 3213, 3216, 3217, 3218, 3219, 6141, 6142, 6143, 7114, 9271
hospitality	hospitality	1221, 1223, 1224, 5434, 5435, 5436, 6231, 6232, 6240, 9273, 9274, 9275, 9279
manual	manual	5111, 5112, 5113,5114, 5119, 5211, 5212, 5213, 5214, 5215, 5216, 5221, 5222, 5223, 5224, 5225, 5231, 5232, 5234, 5235, 5236, 5237, 5250, 5311, 5312, 5313, 5314, 5315, 5316, 5319, 5321, 5322, 5323, 5330, 5411, 5412, 5413, 5414, 5419, 5421, 5422, 5423, 5441, 5442, 5449, 8112, 8113, 8114, 8115, 8116, 8117, 8118, 8119, 8121, 8122, 8123, 8124, 8125, 8126, 8127, 8129, 8131, 8132, 8133, 8134, 8135, 8137, 8139, 8141, 8142, 8143, 8149, 9111, 9119, 9120, 9134, 9139, 9260, 9112
other workers	other workers-non-office based	1211, 1213, 1251, 1252, 2111, 2112, 2113, 2114, 2121, 2122, 2123, 2124, 2126, 2127, 2129, 2141, 2142, 2216, 2436, 2461, 2463, 3111, 3112, 3113, 3114, 3115, 3116, 3119, 3121, 3122, 3413, 3414, 3415, 3416, 3417, 3441, 3442, 3443, 3550, 3565, 3567, 5241, 5242, 5244, 5245, 5249, 6131, 6139, 6211, 7123, 7125, 8221, 8222, 8223, 8229, 9244
	other workers-office based	3533, 3534, 3535, 3536, 3537, 3538, 3539, 3541, 3542, 3543, 3544, 3545, 3546, 3561, 3562, 3563, 3564, 4112, 4113, 4114, 4121, 4122, 4123, 4124, 4129, 4131, 4132, 4135, 4138, 4159, 4161, 4162, 4212, 4214, 4215, 4216, 4217, 6212, 7113,7121, 7122, 7211, 7213, 7214, 7215, 7219, 7220, 9219
personal care	personal care	1253, 6221, 6222
police and protective services	police and protective services	1171, 1172, 1173, 3311, 3312, 3313, 3314, 3315, 3319, 9241, 9242, 9249
retail	retail	1190, 1254, 4133, 4151, 5443, 7111, 7112, 7115, 7124, 7129, 7130, 9251, 9259
sanitation services	sanitation services	1255, 6132, 9132, 9231, 9232, 9233, 9234, 9235, 9236, 9239
social and education	education	2311, 2312, 2314, 2315, 2316, 2317, 2318, 2319, 4213, 6121, 6122, 6123, 6125, 6126
	social care	1184, 1242, 2442, 2443, 2444, 2449, 3231, 3233, 3234, 3235, 3239, 6144, 6145, 6146, 6147, 6148
transport	transport-non public facing	3513, 4134, 8211, 8212, 8231, 8232, 8233, 8234, 8239, 9211
	transport-public facing	3512, 6214, 6215, 6219, 8213, 8214, 8215

Table S2a: Descriptive statistics for all covariates when industry is used as an exposure for self-reported long-COVID (Outcome 1) and reduced function (Outcome 2)-Counts and percentages of individuals

	Long Covid symptoms (Outcome 1)						Reduced function (Outcome 2)							
	No		Yes		Total		Not at all		Yes, a little		Yes, a lot		Total	
	Count	%	Count	%	Count	%	Count	%	Count	%	Count	%	Count	%
**Age bands**														
15-19	21,083	7.2	1,440	4.7	22,523	7.0	587	6.9	648	4.5	204	2.8	1,439	4.7
20-24	15,820	5.4	1,140	3.7	16,960	5.2	436	5.1	553	3.8	151	2.0	1,140	3.7
25-29	20,191	6.9	1,516	5.0	21,707	6.7	541	6.3	721	5.0	254	3.4	1,516	5.0
30-34	24,958	8.5	2,168	7.1	27,126	8.4	673	7.9	1,080	7.4	415	5.6	2,168	7.1
35-39	27,647	9.4	2,911	9.5	30,558	9.4	835	9.7	1,430	9.8	646	8.7	2,911	9.5
40-44	29,224	10.0	3,766	12.3	32,990	10.2	1,080	12.6	1,834	12.6	852	11.5	3,766	12.3
45-49	30,989	10.6	4,032	13.2	35,021	10.8	1,136	13.3	1,950	13.4	946	12.8	4,032	13.2
50-54	34,809	11.9	4,550	14.9	39,359	12.2	1,145	13.4	2,111	14.5	1,296	17.5	4,552	14.9
55-59	38,180	13.0	4,353	14.3	42,533	13.1	1,044	12.2	2,051	14.1	1,259	17.0	4,354	14.3
60-64	39,819	13.6	3,819	12.5	43,638	13.5	901	10.5	1,766	12.1	1,150	15.5	3,817	12.5
65-69	10,503	3.6	848	2.8	11,351	3.5	194	2.3	410	2.8	244	3.3	848	2.8
**Sex**														
Male	135,306	46.1	11,759	38.5	147,065	45.4	3,617	42.2	5,638	38.7	2,504	33.8	11,759	38.5
Female	157,917	53.9	18,784	61.5	176,701	54.6	4,955	57.8	8,916	61.3	4,913	66.2	18,784	61.5
**Ethnicity**														
White	267,663	91.3	28,189	92.3	295,852	91.4	794	92.8	13,414	92.2	6,821	92.0	28,189	92.3
Mixed	4,950	1.7	498	1.6	5,448	1.7	119	1.4	242	1.7	137	1.9	498	1.6
Asian	14,189	4.8	1,247	4.1	15,436	4.8	323	3.8	609	4.2	315	4.3	1,247	4.1
Black	3,459	1.3	305	1.0	3,764	1.2	85	1.0	147	1.0	73	1.0	305	1.0
Other	2,960	1.0	304	1.0	3,264	1.0	91	1.1	142	1.0	71	1.0	304	1.0
**IMD (Index of Multiple Deprivation**														
1st Quartile	44,738	15.3	5,668	18.6	50,406	15.6	1,318	15.4	2,617	18.0	1,734	23.4	5,669	18.6
2nd Quartile	68,677	23.4	7,360	24.1	76,037	23.5	1,936	22.6	3,495	24.0	1,929	26.0	7,360	24.1
3rd Quartile	83,677	28.5	8,398	27.5	92,075	28.4	2,453	28.6	4,046	27.8	1,898	25.6	8,397	27.5
4th Quartile	96,131	32.8	9,117	29.9	105,248	32.5	2,865	33.4	4,396	30.2	1,856	25.0	9,117	29.9
**Government office regions**														

North East	10,033	3.4	1,349	4.4	11,382	3.5	349	4.1	640	4.4	360	4.9	1,349	4.4
North West	32,049	10.9	3,914	12.8	35,963	11.1	1,069	12.5	1,873	12.9	972	13.1	3,914	12.8
Yorkshire & the Humber	23,074	7.9	2,692	8.8	25,766	8.0	743	8.7	1,264	8.7	686	9.3	2,693	8.8
East Midlands	18,474	6.3	2,032	6.7	20,506	6.3	629	7.3	944	6.5	460	6.2	2,033	6.7
West Midlands	21,127	7.2	2,430	8.0	23,557	7.3	683	8.0	1,122	7.7	625	8.4	2,430	8.0
East of England	26,605	9.1	2,704	8.9	29,309	9.1	799	9.3	1,273	8.8	631	8.5	2,703	8.9
London	56,565	19.3	5,190	17.0	61,755	19.1	1,536	17.9	2,492	17.1	1,161	15.7	5,189	17.0
South East	36,248	12.4	3,477	11.4	39,725	12.3	1,035	12.1	1,702	11.7	740	10.0	3,477	11.4
South West	22,150	7.6	2,147	7.0	24,297	7.5	636	7.4	1,054	7.2	458	6.2	2,148	7.0
Northern Ireland	8,378	2.9	838	2.7	9,216	2.9	180	2.1	417	2.9	241	3.3	838	2.7
Scotland	24,138	8.2	2,266	7.4	26,404	8.2	575	6.7	1,050	7.2	641	8.6	2,266	7.4
Wales	14,382	4.9	1,504	4.9	15,886	4.9	338	3.9	723	5.0	442	6.0	1,503	4.9
**Urban/Rural**														
Major urban	110,000	37.5	11,547	37.8	121,547	37.5	3,212	37.5	5,487	37.7	2,848	38.4	11,547	37.8
Urban city or town	122,942	41.9	13,315	43.6	136,257	42.1	3,697	43.1	6,367	43.8	3,250	43.8	13,314	43.6
Rural town	29,107	9.9	2,890	9.5	31,997	9.9	814	9.5	1,410	9.7	667	9.0	2,891	9.5
Rural village	31,174	10.6	2,791	9.1	33,965	10.5	849	9.9	1,290	8.9	652	8.8	2,791	9.1
**Household Size**														
One	38,251	13.1	4,202	13.8	42,453	13.1	909	10.6	1,983	13.6	1,310	17.7	4,202	13.8
Two	109,005	37.2	10,377	34.0	119,382	36.9	2,771	32.3	4,966	34.1	2,640	35.6	10,377	34.0
Three	59,258	20.2	6,298	20.6	65,556	20.3	1,876	21.9	2,919	20.1	1,503	20.3	6,298	20.6
Four	59,824	20.4	6,646	21.8	66,470	20.5	2,098	24.5	3,231	22.2	1,317	17.8	6,646	21.8
Five plus	26,885	9.2	3,020	9.9	29,905	9.2	918	10.7	1,455	10.0	647	8.7	3,020	9.9
**Health conditions**														
No	248,054	84.6	23,727	77.7	271,781	83.9	7,371	86.0	11,588	79.6	4,768	64.3	23,727	77.7
Yes	45,169	15.4	6,816	22.3	51,985	16.1	1,201	14.0	2,966	20.4	2,649	35.7	6,816	22.3
**Total**	**293,223**	**90.6**	**30,543**	**9.4**	**323,766**	**100.0**	**8,572**	**100.0**	**14,554**	**100.0**	**7,417**	**100.0**	**30,543**	**100.0**

Table S2b: Descriptive statistics for all covariates when occupational groups variable is used as an exposure for self-reported long-COVID (Outcome 1) and reduced function (Outcome 2)-Counts and percentages of individuals

	Long Covid symptoms (Outcome 1)						Reduced function (Outcome 2)							
	No		Yes		Total		Not at all		Yes, a little		Yes, a lot		Total	
	Count	%	Count	%	Count	%	Count	%	Count	%	Count	%	Count	%
**Age bands**														
15-19	20,771	8.3	1,415	5.5	22,186	8.0	582	8.1	634	5.2	198	3.1	1,414	5.5
20-24	14,149	5.6	998	3.9	15,147	5.5	393	5.4	481	3.9	124	1.9	998	3.9
25-29	16,607	6.6	1,234	4.8	17,841	6.4	429	5.9	591	4.8	214	3.3	1,234	4.8
30-34	20,492	8.1	1,780	6.9	22,272	8.0	545	7.6	887	7.2	348	5.4	1,780	6.9
35-39	22,596	9.0	2,348	9.1	24,944	9.0	682	9.4	1,144	9.3	522	8.2	2,348	9.1
40-44	23,787	9.5	3,065	11.8	26,852	9.7	841	11.6	1,504	12.3	720	11.3	3,065	11.8
45-49	25,318	10.1	3,323	12.8	28,641	10.3	923	12.8	1,621	13.2	778	12.2	3,322	12.8
50-54	28,866	11.5	3,752	14.5	32,618	11.8	929	12.9	1,723	14.1	1,102	17.2	3,754	14.5
55-59	32,773	13.0	3,744	14.5	36,517	13.2	906	12.5	1,719	14.0	1,120	17.5	3,745	14.5
60-64	36,261	14.4	3,435	13.3	39,696	14.3	812	11.2	1,585	12.9	1,037	16.2	3,434	13.3
65-69	9,979	4.0	794	3.1	10,773	3.9	181	2.5	376	3.1	237	3.7	794	3.1
**Sex**														
Male	114,942	45.7	9,826	38.0	124,768	45.0	3,010	41.7	4,663	38.0	2,152	33.6	9,825	38.0
Female	136,657	54.3	16,062	62.0	152,719	55.0	4,213	58.3	7,602	62.0	4,248	66.4	16,063	62.1
**Ethnicity**														
White	229,676	91.3	23,886	92.3	253,562	91.4	6697	92.7	11,295	92.1	5,894	92.1	23,886	92.3
Mixed	4,265	1.7	428	1.7	4,693	1.7	109	1.5	204	1.7	115	1.8	428	1.7
Asian	12,134	4.8	1,062	4.1	13,196	4.8	274	3.8	518	4.2	270	4.2	1,062	4.1
Black	3,009	1.2	256	1.0	3,265	1.2	69	1.0	126	1.0	61	1.0	256	1.0
Other	2,513	1.0	256	1.0	2,769	1.0	74	1.0	122	1.0	60	0.9	256	1.0
**IMD (Index of Multiple Deprivation**														
1st Quartile	39,010	15.5	4,908	19.0	43,918	15.8	1,114	15.4	2,260	18.4	1,535	24.0	4,909	19.0
2nd Quartile	58,714	23.3	6,292	24.3	65,006	23.4	1,616	22.4	2,989	24.4	1,688	26.4	6,293	24.3
3rd Quartile	71,560	28.4	7,095	27.4	78,655	28.4	2,066	28.6	3,396	27.7	1,631	25.5	7,093	27.4
4th Quartile	82,315	32.7	7,593	29.3	89,908	32.4	2,427	33.6	3,620	29.5	1,546	24.2	7,593	29.3
**Government office regions**														

North East	8,563	3.4	1,124	4.3	9,687	3.5	288	4.0	533	4.4	303	4.7	1,124	4.3
North West	27,026	10.7	3,241	12.5	30,267	10.9	872	12.1	1,540	12.6	829	13.0	3,241	12.5
Yorkshire & the Humber	19,772	7.9	2,264	8.8	22,036	7.9	627	8.7	1,056	8.6	582	9.1	2,265	8.8
East Midlands	15,832	6.3	1,728	6.7	17,560	6.3	548	7.6	792	6.5	389	6.1	1,729	6.7
West Midlands	18,163	7.2	2,033	7.9	20,196	7.3	572	7.9	931	7.6	530	8.3	2,033	7.9
East of England	22,254	8.9	2,230	8.6	24,484	8.8	655	9.1	1,033	8.4	541	8.5	2,229	8.6
London	46,784	18.6	4,276	16.5	51,060	18.4	1,245	17.2	2,049	16.7	982	15.3	4,276	16.5
South East	31,041	12.3	2,919	11.3	33,960	12.2	860	11.9	1,436	11.7	622	9.7	2,918	11.3
South West	19,331	7.7	1,861	7.2	21,192	7.6	559	7.7	902	7.4	401	6.3	1,862	7.2
Northern Ireland	7,431	3.0	738	2.9	8,169	2.9	155	2.2	368	3.0	215	3.4	738	2.9
Scotland	22,263	8.9	2,110	8.2	24,373	8.8	539	7.5	973	7.9	598	9.3	2,110	8.2
Wales	13,139	5.2	1,364	5.3	14,503	5.2	303	4.2	652	5.3	408	6.4	1,363	5.3
**Urban/Rural**														
Major urban	92,337	36.7	9,624	37.2	101,961	36.7	2,640	36.6	4,553	37.1	2,432	38.0	9,625	37.2
Urban city or town	106,614	42.4	11,333	43.8	117,947	42.5	3,131	43.4	5,395	44.0	2,806	43.8	11,332	43.8
Rural town	25,176	10.0	2,494	9.6	27,670	10.0	713	9.9	1,194	9.7	587	9.2	2,494	9.6
Rural village	27,472	10.9	2,437	9.4	29,909	10.8	739	10.2	1,123	9.2	575	9.0	2,437	9.4
**Household Size**														
One	32,671	13.0	3,562	13.8	36,233	13.1	733	10.2	1,671	13.6	1,158	18.1	3,562	13.8
Two	93,306	37.1	8,859	34.2	102,165	36.8	2,327	32.2	4,234	34.5	2,298	35.9	8,859	34.2
Three	50,670	20.1	5,290	20.4	55,960	20.2	1,591	22.0	2,423	19.8	1,276	19.9	5,290	20.4
Four	51,287	20.4	5,627	21.7	56,914	20.5	1,800	24.9	2,723	22.2	1,104	17.3	5,627	21.7
Five plus	23,665	9.4	2,550	9.9	26,215	9.5	772	10.7	1,214	9.9	564	8.8	2,550	9.9
**Health conditions**														
No	210,591	83.7	19,808	76.5	230,399	83.0	6,167	85.4	9,664	78.8	3,976	62.1	19,807	76.5
Yes	41,008	16.3	6,080	23.5	47,088	17.0	1,056	14.6	2,601	21.2	2,424	37.9	6,081	23.5
Total	**251,599**	**90.7**	**25,888**	**9.3**	**277,487**	**100.0**	**7,223**	**100.0**	**12,265**	**100.0**	**6,400**	**100.0**	**25,888**	**100.0**

Table S2c: Descriptive statistics for all covariates when SOC occupations variable is used as an exposure for self-reported long-COVID (Outcome 1) and reduced function (Outcome 2)-Counts and percentages of individuals

	Long Covid symptoms (Outcome 1)						Reduced function (Outcome 2)							
	No		Yes		Total		Not at all		Yes, a little		Yes, a lot		Total	
	Count	%	Count	%	Count	%	Count	%	Count	%	Count	%	Count	%
**Age bands**														
15-19	20,911	7.5	1,430	4.9	22,341	7.3	585	7.2	641	4.7	203	2.9	1,429	4.9
20-24	15,193	5.5	1,089	3.8	16,282	5.3	421	5.2	533	3.9	135	1.9	1,089	3.8
25-29	18,864	6.8	1,432	4.9	20,296	6.6	515	6.3	676	4.9	241	3.4	1,432	4.9
30-34	23,390	8.4	2,045	7.1	25,435	8.3	626	7.7	1,021	7.4	398	5.7	2,045	7.1
35-39	25,890	9.3	2,715	9.4	28,605	9.3	788	9.7	1,328	9.6	599	8.5	2,715	9.4
40-44	27,315	9.8	3,520	12.2	30,835	10.0	999	12.2	1,721	12.5	800	11.4	3,520	12.2
45-49	28,983	10.4	3,807	13.1	32,790	10.7	1,085	13.3	1,831	13.3	890	12.6	3,806	13.1
50-54	32,671	11.7	4,270	14.7	36,941	12.0	1,073	13.1	1,974	14.3	1,225	17.4	4,272	14.7
55-59	36,252	13.0	4,146	14.3	40,398	13.1	1,000	12.3	1,935	14.1	1,212	17.2	4,147	14.3
60-64	38,585	13.9	3,694	12.8	42,279	13.8	879	10.8	1,711	12.4	1,102	15.6	3,692	12.7
65-69	10,329	3.7	832	2.9	11,161	3.6	192	2.4	399	2.9	241	3.4	832	2.9
**Sex**														
Male	127,779	45.9	11,075	38.2	138,854	45.2	3,422	41.9	5,292	38.4	2,360	33.5	11,074	38.2
Female	150,604	54.1	17,905	61.8	168,509	54.8	4,741	58.1	8,478	61.6	4,686	66.5	17,905	61.8
**Ethnicity**														
White	254,277	91.3	26,748	92.3	281,025	91.4	7586	92.9	12,679	92.1	6,482	92.0	26,747	92.3
Mixed	4,702	1.7	477	1.7	5,179	1.7	116	1.4	231	1.7	130	1.9	477	1.7
Asian	13,334	4.8	1,181	4.1	14,515	4.7	300	3.7	583	4.2	298	4.2	1,181	4.1
Black	3,272	1.2	286	1.0	3,558	1.2	76	0.9	141	1.0	69	1.0	286	1.0
Other	2,796	1.0	288	1.0	3,084	1.0	85	1.0	136	1.0	67	1.0	288	1.0
**IMD (Index of Multiple Deprivation**														
1st Quartile	42,574	15.3	5,393	18.6	47,967	15.6	1,241	15.2	2,502	18.2	1,651	23.4	5,394	18.6
2nd Quartile	65,160	23.4	7,007	24.2	72,167	23.5	1,840	22.5	3,320	24.1	1,847	26.2	7,007	24.2
3rd Quartile	79,472	28.6	7,958	27.5	87,430	28.5	2,341	28.7	3,815	27.7	1,800	25.6	7,956	27.5

4th Quartile	91,177	32.8	8,622	29.8	99,799	32.5	2,741	33.6	4,133	30.0	1,748	24.8	8,622	29.8
**Government office regions**														
North East	9,540	3.4	1,279	4.4	10,819	3.5	325	4.0	605	4.4	349	5.0	1,279	4.4
North West	30,258	10.9	3,678	12.7	33,936	11.0	1,004	12.3	1,761	12.8	913	13.0	3,678	12.7
Yorkshire & the Humber	21,969	7.9	2,571	8.9	24,540	8.0	713	8.7	1,206	8.8	653	9.3	2,572	8.9
East Midlands	17,544	6.3	1,938	6.7	19,482	6.3	603	7.4	899	6.5	437	6.2	1,939	6.7
West Midlands	20,160	7.2	2,320	8.0	22,480	7.3	658	8.1	1,071	7.8	591	8.4	2,320	8.0
East of England	25,298	9.1	2,577	8.9	27,875	9.1	754	9.2	1,211	8.8	611	8.7	2,576	8.9
London	52,959	19.0	4,886	16.9	57,845	18.8	1,447	17.7	2,346	17.0	1,092	15.5	4,885	16.9
South East	34,384	12.4	3,282	11.3	37,666	12.3	985	12.1	1,611	11.7	685	9.7	3,281	11.3
South West	21,195	7.6	2,021	7.0	23,216	7.6	614	7.5	971	7.1	437	6.2	2,022	7.0
Northern Ireland	7,851	2.8	785	2.7	8,636	2.8	171	2.1	384	2.8	230	3.3	785	2.7
Scotland	23,428	8.4	2,205	7.6	25,633	8.3	566	6.9	1,014	7.4	625	8.9	2,205	7.6
Wales	13,797	5.0	1,438	5.0	15,235	5.0	323	4.0	691	5.0	423	6.0	1,437	5.0
**Urban/Rural**														
Major urban	103,478	37.2	10,918	37.7	114,396	37.2	3,032	37.1	5,192	37.7	2,694	38.2	10,918	37.7
Urban city or town	117,243	42.1	12,617	43.5	129,860	42.3	3,521	43.1	6,003	43.6	3,092	43.9	12,616	43.5
Rural town	27,806	10.0	2,790	9.6	30,596	10.0	794	9.7	1,350	9.8	646	9.2	2,790	9.6
Rural village	29,856	10.7	2,655	9.2	32,511	10.6	816	10.0	1,225	8.9	614	8.7	2,655	9.2
**Household Size**														
One	36,288	13.0	3,975	13.7	40,263	13.1	857	10.5	1,867	13.6	1,251	17.8	3,975	13.7
Two	103,553	37.2	9,881	34.1	113,434	36.9	2,632	32.2	4,727	34.3	2,521	35.8	9,880	34.1
Three	56,126	20.2	5,972	20.6	62,098	20.2	1,800	22.1	2,754	20.0	1,418	20.1	5,972	20.6
Four	56,783	20.4	6,303	21.8	63,086	20.5	2,012	24.7	3,056	22.2	1,235	17.5	6,303	21.8
Five plus	25,633	9.2	2,849	9.8	28,482	9.3	862	10.6	1,366	9.9	621	8.8	2,849	9.8
**Health conditions**														
No	234,681	84.3	22,422	77.4	257,103	83.7	7,010	85.9	10,935	79.4	4,476	63.5	22,421	77.4
Yes	43,702	15.7	6,558	22.6	50,260	16.4	1,153	14.1	2,835	20.6	2,570	36.5	6,558	22.6
**Total**	**278,383**	**90.6**	**28,980**	**9.4**	**307,363**	**100.0**	**8,163**	**100.0**	**13,770**	**100.0**	**7,046**	**100.0**	**28,979**	**100.0**

Table S3: Outcome Missingness patterns of all covariates used for adjustment purposes

	Self-Reported Long-COVID				
**Covariates**					
**Age categories**	**No**	**Yes**	**Total (missing excluded)**	**Missing**	**Total**
15-19	21,187	1,451	22,638	64	22,702
%	7.10	4.67	6.87	6.77	6.87
20-24	16,089	1,158	17,247	142	17,389
%	5.39	3.73	5.24	15.01	5.26
25-29	20,552	1,547	22,099	118	22,217
%	6.89	4.98	6.71	12.47	6.73
30-34	25,424	2,212	27,636	129	27,765
%	8.52	7.12	8.39	13.64	8.41
35-39	28,213	2,965	31,178	74	31,252
%	9.46	9.55	9.47	7.82	9.46
40-44	29,884	3,832	33,716	83	33,799
%	10.02	12.34	10.24	8.77	10.23
45-49	31,661	4,110	35,771	61	35,832
%	10.61	13.24	10.86	6.45	10.85
50-54	35,524	4,636	40,160	99	40,259
%	11.91	14.93	12.19	10.47	12.19
55-59	38,842	4,410	43,252	82	43,334
%	13.02	14.20	13.13	8.67	13.12
60-64	40,300	3,869	44,169	73	44,242
%	13.51	12.46	13.41	7.72	13.4
65-69	10,615	858	11,473	21	11,494
%	3.56	2.76	3.48	2.22	3.48
**Total**	**298,291**	**31,048**	**329,339**	**946**	**330,285**
**%**	**100.00**	**100.00**	**100.00**	**100.00**	**100**
**Sex**					
Male	137,895	11,998	149,893	470	150,363
%	46.23	38.64	45.51	49.68	45.53
Female	160,396	19,050	179,446	476	179,922
%	53.77	61.36	54.49	50.32	54.47
**Total**	**298,291**	**31,048**	**329,339**	**946**	**330,285**
**%**	**100**	**100**	**100**	**100**	**100**
**Ethnicity**					
White	272,298	28,655	300,953	833	301,786
%	91.29	92.29	91.38	88.05	91.37
Mixed	5,041	508	5,549	27	5,576
%	1.69	1.64	1.68	2.85	1.69
Asian	14,408	1,266	15,674	44	15,718
%	4.83	4.08	4.76	4.65	4.76
Black	3,533	310	3,843	22	3,865
%	1.18	1	1.17	2.33	1.17
Other	3,009	309	3,318	16	3,334
%	1.01	1	1.01	1.69	1.01

.	2	0	2	4	6
%	0	0	0.00	0.42	0
**Total**	**298,291**	**31,048**	**329,339**	**946**	**330,285**
**%**	**100**	**100**		**100**	**100**
**Deprivation (Quintiles)**					
1st Quartile	45,474	5,762	51,236	149	51,385
%	15.24	18.56	15.56	15.75	15.56
2nd Quartile	69,967	7,477	77,444	268	77,712
%	23.46	24.08	23.51	28.33	23.53
3rd Quartile	85,177	8,540	93,717	250	93,967
%	28.56	27.51	28.46	26.43	28.45
4th Quartile	97,673	9,269	106,942	279	107,221
%	32.74	29.85	32.47	29.49	32.46
**Total**	**298,291**	**31,048**	**329,339**	**946**	**330,285**
**%**	**100**	**100**		**100**	**100**
**UK Region**					
North East	10,169	1,368	11,537	18	11,555
%	3.41	4.41	3.50	1.9	3.5
North West	32,607	3,974	36,581	66	36,647
%	10.93	12.8	11.11	6.98	11.1
Yorkshire & the Humber	23,436	2,739	26,175	20	26,195
%	7.86	8.82	7.95	2.11	7.93
East Midlands	18,841	2,087	20,928	25	20,953
%	6.32	6.72	6.35	2.64	6.34
West Midlands	21,628	2,472	24,100	38	24,138
%	7.25	7.96	7.32	4.02	7.31
East of England	27,113	2,763	29,876	59	29,935
%	9.09	8.9	9.07	6.24	9.06
London	57,753	5,277	63,030	278	63,308
%	19.36	17	19.14	29.39	19.17
South East	36,803	3,532	40,335	78	40,413
%	12.34	11.38	12.25	8.25	12.24
South West	22,688	2,187	24,875	61	24,936
%	7.61	7.04	7.55	6.45	7.55
Northern Ireland	8,460	848	9,308	31	9,339
%	2.84	2.73	2.83	3.28	2.83
Scotland	24,275	2,280	26,555	120	26,675
%	8.14	7.34	8.06	12.68	8.08
Wales	14,518	1,521	16,039	152	16,191
%	4.87	4.9	4.87	16.07	4.9
**Total**	**298,291**	**31,048**	**329,339**	**946**	**330,285**
**%**	**100**	**100**	**100.00**	**100**	**100**
**Urban or Rural**					
Major urban	112,006	11,720	123,726	389	124,115
%	37.55	37.75	37.57	41.12	37.58
Urban city or town	124,996	13,560	138,556	345	138,901
%	41.9	43.67	42.07	36.47	42.05
Rural town	29,599	2,938	32,537	97	32,634

%	9.92	9.46	9.88	10.25	9.88
Rural village	31,690	2,830	34,520	115	34,635
%	10.62	9.11	10.48	12.16	10.49
**Total**	**298,291**	**31,048**	**329,339**	**946**	**330,285**
**%**	**100**	**100**	**100.00**	**100**	**100**
**Household Size**					
1	38,842	4,269	43,111	129	43,240
%	13.02	13.75	13.09	13.64	13.09
2	110,725	10,543	121,268	339	121,607
%	37.12	33.96	36.82	35.84	36.82
3	60,275	6,386	66,661	185	66,846
%	20.21	20.57	20.24	19.56	20.24
4	61,022	6,789	67,811	196	68,007
%	20.46	21.87	20.59	20.72	20.59
5+	27,427	3,061	30,488	97	30,585
%	9.19	9.86	9.26	10.25	9.26
**Total**	**298,291**	**31,048**	**329,339**	**946**	**330,285**
**%**	**100**	**100**	**100.00**	**100**	**100**
**Health conditions**					
No	252,641	24,163	276,804	789	277,593
%	84.7	77.82	84.05	83.4	84.05
Yes	45,650	6,885	52,535	134	52,669
%	15.3	22.18	15.95	14.16	15.95
.	0	0	0	23	23
%	**0**	**0**	**0.00**	**2.43**	**0.01**
**Total**	**298,291**	**31,048**	**329,339**	**946**	**330,285**

Table S4: Outcome Missingness patterns of all exposures used

	**Self-Reported Long-COVID**				
**Exposures**					
**Industries (SIC)**	**No**	**Yes**	**Total (No missing)**	**Missing**	**Total**
Teaching and education	25,568	3,708	29,276	60	29,336
%	8.72	12.14	9.04	6.56	9.04
Health care	22,569	2,762	25,331	71	25,402
%	7.70	9.04	7.82	7.76	7.82
Social care	5,959	845	6,804	22	6,826
%	2.03	2.77	2.10	2.40	2.10
Transport (incl. storage, logistic)	7,217	801	8,018	23	8,041
%	2.46	2.62	2.48	2.51	2.48
Retail sector (incl.wholesale)	12,669	1,413	14,082	41	14,123
%	4.32	4.63	4.35	4.48	4.35
Hospitality (e.g. hotel restaurants)	4,078	426	4,504	9	4,513
%	1.39	1.39	1.39	0.98	1.39
Food production, agriculture, farming)	3,517	349	3,866	16	3,882
%	1.20	1.14	1.19	1.75	1.20

Personal services (e.g hairdressers)	1,890	191	2,081	5	2,086
%	0.64	0.63	0.64	0.55	0.64
Information technology and communication	15,795	1,215	17,010	49	17,059
%	5.39	3.98	5.25	5.36	5.25
Financial services inc. insurance	17,761	1,424	19,185	52	19,237
%	6.06	4.66	5.93	5.68	5.92
Manufacturing or construction	20,206	2,034	22,240	55	22,295
%	6.89	6.66	6.87	6.01	6.87
Civil service or Local Goverbment	15,027	1,729	16,756	53	16,809
%	5.12	5.66	5.18	5.79	5.18
Armed forces	827	66	893	4	897
%	0.28	0.22	0.28	0.44	0.28
Arts,Entertainment or Recreation	4,530	416	4,946	19	4,965
%	1.54	1.36	1.53	2.08	1.53
Other occupation sect	30,576	2,816	33,392	113	33,505
%	10.43	9.22	10.31	12.35	10.32
Not working	105,034	10,348	115,382	323	115,705
%	35.82	33.88	35.64	35.30	35.64
**Total**	**293,223**	**30,543**	**323,766**	**915**	**324,681**
**%**	**100**	**100.00**	**100.00**	**100.00**	**100.00**
**Occupations (Constructed by authors)**					
education	12,103	1,883	13,986	43	14,029
%	4.81	7.27	5.04	5.22	5.04
food processing	1,167	138	1,305	8	1,313
%	0.46	0.53	0.47	0.97	0.47
healthcare-office based	862	94	956	1	957
%	0.34	0.36	0.34	0.12	0.34
healthcare-patient contact	11,787	1,370	13,157	32	13,189
%	4.68	5.29	4.74	3.88	4.74
Hospitality	2,390	310	2,700	5	2,705
%	0.95	1.20	0.97	0.61	0.97
Manual	11,520	1,107	12,627	41	12,668
%	4.58	4.28	4.55	4.98	4.55
other workers-non-office based	12,160	1,151	13,311	39	13,350
%	4.83	4.45	4.80	4.73	4.80
other workers-office based	73,628	6,741	80,369	254	80,623
%	29.26	26.04	28.96	30.83	28.97
personal care	334	37	371	1	372
%	0.13	0.14	0.13	0.12	0.13
police and protective services	3,235	418	3,653	19	3,672
%	1.29	1.61	1.32	2.31	1.32
Retail	5,821	655	6,476	20	6,496
%	2.31	2.53	2.33	2.43	2.33
sanitation services	1,697	204	1,901	1	1,902
%	0.67	0.79	0.69	0.12	0.68
social care	7,006	1,093	8,099	27	8,126
%	2.78	4.22	2.92	3.28	2.92
transport-nonpublic facing	3,202	360	3,562	10	3,572

	1.27	1.39	1.28		1.21	1.28
transport-public facing	970	118	1,088		1	1,089
%	0.39	0.46	0.39		0.12	0.39
Not working	103,717	10,209	113,926		322	114,248
%	41.22	39.44	41.06		39.08	41.05
**Total**	**251,599**	**25,888**	**277,487**		**824**	**278,311**
**%**	**100**	**100**	**100**		**100**	**100**
**Occupations (SOC)**						
Managers, directors and senior officials	20,812	2,126	22,938		50	22,988
%	7.48	7.34	7.46		5.79	7.46
Professional occupations	54,576	5,476	60,052		170	60,222
%	19.60	18.90	19.54		19.70	19.54
Associate professional and technical	32,017	3,263	35,280		102	35,382
%	11.50	11.26	11.48		11.82	11.48
Admin and secretarial	22,839	2,347	25,186		59	25,245
%	8.20	8.10	8.19		6.84	8.19
Skilled trades	12,000	1,168	13,168		37	13,205
%	4.31	4.03	4.28		4.29	4.28
Caring, leisure and other service	11,425	1,956	13,381		52	13,433
%	4.10	6.75	4.35		6.03	4.36
Sales and customer service	7,805	934	8,739		27	8,766
%	2.80	3.22	2.84		3.13	2.84
Process plant and machine operatives	5,937	658	6,595		19	6,614
%	2.13	2.27	2.15		2.20	2.15
Elementary	7,255	843	8,098		25	8,123
%	2.61	2.91	2.63		2.90	2.64
Not working	103,717	10,209	113,926		322	114,248
%	37.26	35.23	37.07		37.31	37.07
**Total**	**278,383**	**28,980**	**307,363**		**863**	**308,226**
**%**	**100.00**	**100.00**	**100.00**		**100.00**	**100.00**

Table S5. Working and not working group by prevalence of health conditions (a: Long-Covid sample, b: reduced function sample)

**Health condition**									
**(a)**	**Industry (SIC)**			**Occupation**			**SOC major group**		
**Health conditions**	**Working**	**Non-working**	**TOTAL**	**Working**	**Non-working**	**TOTAL**	**Working**	**Non-working**	**TOTAL**
**No**	183 265	88 516	**271 781**	143 161	87 238	**230 399**	169 865	87 238	**257 103**
**%**	87.95	76.72	**83.94**	87.53	76.57	**83.03**	87.81	76.57	**83.65**
**Yes**	25 119	26 866	**51 985**	20 400	26 688	**47 088**	23 572	26 688	**50 260**
**%**	12.05	23.28	**16.06**	12.47	23.43	**16.97**	12.19	23.43	**16.35**
**TOTAL**	208 384	115 382	**323 766**	163 561	113 926	**277 487**	193 437	113 926	**307 363**
**%**	64.36	35.64	**100.00**	58.94	41.06	**100.00**	62.93	37.07	**100.00**

**Health condition**									
**(b)**	**Industry (SIC)**			**Occupation**			**SOC major group**		
**Health conditions**	**Working**	**Non-working**	**TOTAL**	**Working**	**Non-working**	**TOTAL**	**Working**	**Non-working**	**TOTAL**
**No**	16 702	7 025	**23 727**	12 894	6 913	**19 807**	15 508	6 913	**22 421**
	82.70	67.89	**77.68**	82.24	67.71	**76.51**	82.62	67.71	**77.37**
**Yes**	3 493	3 323	**6 816**	2 785	3 296	**6 081**	3 262	3 296	**6 558**
	17.30	32.11	**22.32**	17.76	32.29	**23.49**	17.38	32.29	**22.63**
**TOTAL**	20 195	10 348	**30 543**	15 679	10 209	**25 888**	18 770	10 209	**28 979**
	100.00	100.00	**100.00**	100.00	100.00	**100.00**	100.00	100.00	**100.00**

Table S6. Working and not working group across industries by prevalence of long-COVID symptoms (a) and reduced function (b) and age sex and health conditions

a.

	Exposure groups	Industry (SIC)																	
		Working						Not working						Total					
		Count			%			Count			%			Count			%		
**Outcome**	**Self-reported long-COVID**	**No**	**Yes**	**Total**	**No**	**Yes**	**Total**	**No**	**Yes**	**Total**	**No**	**Yes**	**Total**	**No**	**Yes**	**Total**	**No**	**Yes**	**Total**
Age bands	15-19	1 564	126	1690	92.5	7.5	100.0	19519	1314	20833	93.69	6.31	100.00	21083	1440	22523	93.6	6.4	100.0
	20-24	7 944	605	8549	92.9	7.1	100.0	7876	535	8411	93.6	6.4	100.0	15820	1140	16960	93.3	6.7	100.0
	25-29	15 880	1211	17091	92.9	7.1	100.0	4311	305	4616	93.4	6.6	100.0	20191	1516	21707	93.0	7.0	100.0
	30-34	19 822	1654	21476	92.3	7.7	100.0	5136	514	5650	90.9	9.1	100.0	24958	2168	27126	92.0	8.0	100.0
	35-39	22 124	2215	24339	90.9	9.1	100.0	5523	696	6219	88.8	11.2	100.0	27647	2911	30558	90.5	9.5	100.0
	40-44	23 978	2965	26943	89.0	11.0	100.0	5246	801	6047	86.8	13.3	100.0	29224	3766	32990	88.6	11.4	100.0
	45-49	25 282	3192	28474	88.8	11.2	100.0	5707	840	6547	87.2	12.8	100.0	30989	4032	35021	88.5	11.5	100.0
	50-54	27 137	3421	30558	88.8	11.2	100.0	7672	1129	8801	87.2	12.8	100.0	34809	4550	39359	88.4	11.6	100.0
	55-59	24 935	2818	27753	89.9	10.2	100.0	13245	1535	14780	89.6	10.4	100.0	38180	4353	42533	89.8	10.2	100.0
	60-64	16 901	1780	18681	90.5	9.5	100.0	22918	2039	24957	91.8	8.2	100.0	39819	3819	43638	91.2	8.8	100.0
	65-69	2 622	208	2830	92.7	7.4	100.0	7881	640	8521	92.5	7.5	100.0	10503	848	11351	92.5	7.5	100.0
Sex	Male	92 524	8323	100847	91.8	8.3	100.0	42782	3436	46218	92.6	7.4	100.0	135306	11759	147065	92.0	8.0	100.0
	Female	95 665	11872	107537	89.0	11.0	100.0	62252	6912	69164	90.0	10.0	100.0	157917	18784	176701	89.4	10.6	100.0
Health Condition	No	166 564	16701	183265	90.9	9.1	100.0	81490	7026	88516	92.1	7.9	100.0	248054	23727	271781	91.3	8.7	100.0
	Yes	21 625	3494	25119	86.1	13.9	100.0	23544	3322	26866	87.6	12.4	100.0	45169	6816	51985	86.9	13.1	100.0
**Total**		**188,189**	**20195**	**208384**	**90.3**	**9.7**	**100.0**	**105034**	**10348**	**115382**	**91.0**	**9.0**	**100.0**	**293223**	**30543**	**323766**	**90.6**	**9.4**	**100.0**

b.

		Working								Not working							
		Count				%				Count				%			
**Outcome**	**Reduced function**	**No**	**Yes, a little**	**Yes, a lot**	**Total**	**No**	**Yes, a little**	**Yes, a lot**	**Total**	**No**	**Yes, a little**	**Yes, a lot**	**Total**	**No**	**Yes, a little**	**Yes, a lot**	**Total**
Age bands	15-19	51	55	20	126	40.48	43.65	15.87	100	536	593	184	1313	40.82	45.16	14.01	100
	20-24	236	297	72	605	39.01	49.09	11.9	100	200	256	79	535	37.38	47.85	14.77	100
	25-29	445	588	178	1211	36.75	48.55	14.7	100	96	133	76	305	31.48	43.61	24.92	100
	30-34	530	834	290	1654	32.04	50.42	17.53	100	143	246	125	514	27.82	47.86	24.32	100
	35-39	658	1122	435	2215	29.71	50.65	19.64	100	177	308	211	696	25.43	44.25	30.32	100
	40-44	904	1468	593	2965	30.49	49.51	20	100	176	366	259	801	21.97	45.69	32.33	100
	45-49	960	1574	658	3192	30.08	49.31	20.61	100	176	376	288	840	20.95	44.76	34.29	100
	50-54	936	1678	808	3422	27.35	49.04	23.61	100	209	433	488	1130	18.50	38.32	43.19	100
	55-59	753	1379	687	2819	26.71	48.92	24.37	100	291	672	572	1535	18.96	43.78	37.26	100
	60-64	442	859	477	1778	24.86	48.31	26.83	100	459	907	673	2039	22.51	44.48	33.01	100
	65-69	44	118	46	208	21.15	56.73	22.12	100	150	292	198	640	23.44	45.63	30.94	100
Sex	Male	2,670	4140	1513	8323	32.08	49.74	18.18	100	947	1498	991	3436	27.56	43.6	28.84	100
	Female	3,289	5832	2751	11872	27.70	49.12	23.17	100	1,666	3084	2162	6912	24.10	44.62	31.28	100
Health Condition	No	5,219	8317	3166	16702	31.25	49.8	18.96	100	2,152	3271	1602	7025	30.63	46.56	22.8	100
	Yes	740	1655	1098	3493	29.51	49.38	31.43	100	461	1311	1551	3323	13.87	39.45	46.67	100
**Total**		**5,959**	**9972**	**4264**	**20195**	**29.51**	**49.38**	**21.11**	**100**	**2,613**	**4582**	**3153**	**10348**	**25.25**	**44.28**	**30.47**	**100**

Table S7. Working and not working group across occupations by prevalence of long-COVID symptoms and age, sex and health conditions

a.

	**Exposure groups**	**Occupations**																	
		**Working**						**Not working**						**Total**					
		**Count**			**%**			**Count**			**%**			**Count**			**%**		
**Outcome**	**Self-reported long-COVID**	**No**	**Yes**	**Total**	**No**	**Yes**	**Total**	**No**	**Yes**	**Total**	**No**	**Yes**	**Total**	**No**	**Yes**	**Total**	**No**	**Yes**	**Total**
Age bands	15-19	1 325	107	1 432	92.5	7.5	100.0	19 446	1308	20 754	93.7	6.3	100.0	20 771	1415	22 186	93.6	6.4	100.0
	20-24	6 385	469	6 854	93.2	6.8	100.0	7 764	529	8 293	93.6	6.4	100.0	14 149	998	15 147	93.4	6.6	100.0
	25-29	12 418	935	13 353	93.0	7.0	100.0	4 189	299	4 488	93.3	6.7	100.0	16 607	1234	17 841	93.1	6.9	100.0
	30-34	15 467	1273	16 740	92.4	7.6	100.0	5 025	507	5 532	90.8	9.2	100.0	20 492	1780	22 272	92.0	8.0	100.0
	35-39	17 201	1665	18 866	91.2	8.8	100.0	5 395	683	6 078	88.8	11.2	100.0	22 596	2348	24 944	90.6	9.4	100.0
	40-44	18 690	2299	20 989	89.1	11.0	100.0	5 097	766	5 863	86.9	13.1	100.0	23 787	3065	26 852	88.6	11.4	100.0
	45-49	19 756	2499	22 255	88.8	11.2	100.0	5 562	824	6 386	87.1	12.9	100.0	25 318	3323	28 641	88.4	11.6	100.0
	50-54	21 355	2639	23 994	89.0	11.0	100.0	7 511	1113	8 624	87.1	12.9	100.0	28 866	3752	32 618	88.5	11.5	100.0
	55-59	19 711	2229	21 940	89.8	10.2	100.0	13 062	1515	14 577	89.6	10.4	100.0	32 773	3744	36 517	89.7	10.3	100.0
	60-64	13 454	1409	14 863	90.5	9.5	100.0	22 807	2026	24 833	91.8	8.2	100.0	36 261	3435	39 696	91.3	8.7	100.0
	65-69	2 120	155	2 275	93.2	6.8	100.0	7 859	639	8 498	92.5	7.5	100.0	9 979	794	10 773	92.6	7.4	100.0
Sex	Male	72 716	6444	79 160	91.9	8.1	100.0	42 226	3382	45 608	92.6	7.4	100.0	114 942	9826	124 768	92.1	7.9	100.0
	Female	75 166	9235	84 401	89.1	10.9	100.0	61 491	6827	68 318	90.0	10.0	100.0	136 657	16062	152 719	89.5	10.5	100.0
Health Condition	No	130 267	12894	143 161	91.0	9.0	100.0	80 324	6914	87 238	92.1	7.9	100.0	210 591	19808	230 399	91.4	8.6	100.0
	Yes	17 615	2785	20 400	86.4	13.7	100.0	23 393	3295	26 688	87.7	12.4	100.0	41 008	6080	47 088	87.1	12.9	100.0
**Total**		**147 882**	**15 679**	**163 561**	**90.4**	**9.6**	**100.0**	**103 717**	**10 209**	**113 926**	**91.0**	**9.0**	**100.0**	**251 599**	**25 888**	**277 487**	**90.7**	**9.3**	**100.0**

b.

		**Working**								**Not working**							
		**Count**				**%**				**Count**				**%**			
**Outcome**	**Reduced function**	**No**	**Yes, a little**	**Yes, a lot**	**Total**	**No**	**Yes, a little**	**Yes, a lot**	**Total**	**No**	**Yes, a little**	**Yes, a lot**	**Total**	**No**	**Yes, a little**	**Yes, a lot**	**Total**
Age bands	15-19	47	46	14	107	43.93	42.99	13.08	100	535	588	184	1 307	40.93	44.99	14.08	100
	20-24	195	226	48	469	41.58	48.19	10.23	100	198	255	76	529	37.43	48.2	14.37	100
	25-29	334	462	139	935	35.72	49.41	14.87	100	95	129	75	299	31.77	43.14	25.08	100
	30-34	403	644	226	1 273	31.66	50.59	17.75	100	142	243	122	507	28.01	47.93	24.06	100
	35-39	509	843	313	1 665	30.57	50.63	18.8	100	173	301	209	683	25.33	44.07	30.6	100
	40-44	677	1 151	471	2 299	29.45	50.07	20.49	100	164	353	249	766	21.41	46.08	32.51	100
	45-49	752	1 252	494	2 498	30.10	50.12	19.78	100	171	369	284	824	20.75	44.78	34.47	100
	50-54	726	1 296	618	2 640	27.50	49.09	23.41	100	203	427	484	1 114	18.22	38.33	43.45	100
	55-59	623	1 056	551	2 230	27.94	47.35	24.71	100	283	663	569	1 515	18.68	43.76	37.56	100
	60-64	356	684	368	1 408	25.28	48.58	26.14	100	456	901	669	2 026	22.51	44.47	33.02	100
	65-69	31	84	40	155	20.00	54.19	25.81	100	150	292	197	639	23.47	45.7	30.83	100
Sex	Male	2 081	3 189	1 173	6 443	32.30	49.50	18.21	100	929	1474	979	3 382	27.47	43.58	28.95	100
	Female	2 572	4 555	2 109	9 236	27.85	49.32	22.83	100	1 641	3047	2139	6 827	24.04	44.63	31.33	100
Health Condition	No	4 052	6 440	2 402	12 894	31.43	49.95	18.63	100	2 115	3224	1574	6 913	30.59	46.64	22.77	100
	Yes	601	1 304	880	2785	29.68	49.39	31.60	100	455	1297	1544	3 296	13.80	39.35	46.84	100
**Total**		**4,653**	**7744**	**3282**	**15679**	**29.68**	**49.39**	**20.93**	**100**	**2,570**	**4521**	**3118**	**10209**	**25.17**	**44.28**	**30.54**	**100**

Table S8. Working and not working group across major occupational groups by prevalence of long-COVID symptoms and age, sex and health conditions

a.

	Exposure groups	Major occupational groups (SOC)																	
		Working						Not working						Total					
		Count			%			Count			%			Count			%		
**Outcome**	**Self-reported long-COVID**	**No**	**Yes**	**Total**	**No**	**Yes**	**Total**	**No**	**Yes**	**Total**	**No**	**Yes**	**Total**	**No**	**Yes**	**Total**	**No**	**Yes**	**Total**
Age bands	15-19	1 465	122	1 587	92.3	7.7	100.0	19 446	1 308	20 754	93.7	6.3	100.0	20 911	1 430	22 341	93.6	6.4	100.0
	20-24	7 429	560	7 989	93.0	7.0	100.0	7 764	529	8 293	93.6	6.4	100.0	15 193	1 089	16 282	93.3	6.7	100.0
	25-29	14 675	1 133	15 808	92.8	7.2	100.0	4 189	299	4 488	93.3	6.7	100.0	18 864	1 432	20 296	92.9	7.1	100.0
	30-34	18 365	1 538	19 903	92.3	7.7	100.0	5 025	507	5 532	90.8	9.2	100.0	23 390	2 045	25 435	92.0	8.0	100.0
	35-39	20 95	2 032	22 527	91.0	9.0	100.0	5 395	683	6 078	88.8	11.2	100.0	25 890	2 715	28 605	90.5	9.5	100.0
	40-44	22 218	2 754	24 972	89.0	11.0	100.0	5 097	766	5 863	86.9	13.1	100.0	27 315	3 520	30 835	88.6	11.4	100.0
	45-49	23 421	2 983	26 404	88.7	11.3	100.0	5 562	824	6 386	87.1	12.9	100.0	28 983	3 807	32 790	88.4	11.6	100.0
	50-54	25 160	3 157	28 317	88.9	11.2	100.0	7 511	1 113	8 624	87.1	12.9	100.0	32 671	4 270	36 941	88.4	11.6	100.0
	55-59	23 190	2 631	25 821	89.8	10.2	100.0	13 062	1 515	14 577	89.6	10.4	100.0	36 252	4 146	40 398	89.7	10.3	100.0
	60-64	15 778	1 668	17 446	90.4	9.6	100.0	22 807	2 026	24 833	91.8	8.2	100.0	38 585	3 694	42 279	91.3	8.7	100.0
	65-69	2 470	193	2 663	92.8	7.3	100.0	7 859	639	8 498	92.5	7.5	100.0	10 329	832	11 161	92.5	7.5	100.0
Sex	Male	85 553	7 693	93 246	91.8	8.3	100.0	42 226	3 382	45 608	92.6	7.4	100.0	127 779	11 075	138 854	92.0	8.0	100.0
	Female	89 113	11 078	100 191	88.9	11.1	100.0	61 491	6 827	68 318	90.0	10.0	100.0	150 604	17 905	168 509	89.4	10.6	100.0
Health Condition	No	154 357	15 508	169 865	90.9	9.1	100.0	80 324	6 914	87 238	92.1	7.9	100.0	234 681	22 422	257 103	91.3	8.7	100.0
	Yes	20 309	3 263	23 572	86.2	13.8	100.0	23 393	3 295	26 688	87.7	12.4	100.0	43 702	6 558	50 260	87.0	13.0	100.0
**Total**		**174 666**	**18 771**	**193 437**	**90.30**	**9.7**	**100**	**103 717**	**10 209**	**113 926**	**91.0**	**9.0**	**100.0**	**278 383**	**28 980**	**307 363**	**90.6**	**9.4**	**100.0**

b.

		Working								Not working							
		Count			Total	%			Total	Count			Total	%			Total
**Outcome**	**Reduced function**	**No**	**Yes, a little**	**Yes, a lot**		**No**	**Yes, a little**	**Yes, a lot**		**No**	**Yes, a little**	**Yes, a lot**		**No**	**Yes, a little**	**Yes, a lot**	
Age bands	15-19	50	53	19	122	40.98	43.44	15.57	100	535	588	184	1 307	40.93	44.99	14.08	100
	20-24	223	278	59	560	39.82	49.64	10.54	100	198	255	76	529	37.43	48.2	14.37	100
	25-29	420	547	166	1133	37.07	48.28	14.65	100	95	129	75	299	31.77	43.14	25.08	100
	30-34	484	778	276	1538	31.47	50.59	17.95	100	142	243	122	507	28.01	47.93	24.06	100
	35-39	615	1 027	390	2032	30.27	50.54	19.19	100	173	301	209	683	25.33	44.07	30.6	100
	40-44	835	1 368	551	2754	30.32	49.67	20.01	100	164	353	249	766	21.41	46.08	32.51	100
	45-49	914	1 462	606	2982	30.65	49.03	20.32	100	171	369	284	824	20.75	44.78	34.47	100
	50-54	870	1 547	741	3158	27.55	48.99	23.46	100	203	427	484	1 114	18.22	38.33	43.45	100
	55-59	717	1 272	643	2632	27.24	48.33	24.43	100	283	663	569	1 515	18.68	43.76	37.56	100
	60-64	423	810	433	1666	25.39	48.62	25.99	100	456	901	669	2 026	22.51	44.47	33.02	100
	65-69	42	107	44	193	21.76	55.44	22.8	100	150	292	197	639	23.47	45.7	30.83	100
Sex	Male	2 493	3 818	1 381	7692	32.41	49.64	17.95	100	929	1 474	979	3 382	27.47	43.58	28.95	100
	Female	3 100	5 431	2 547	11078	27.98	49.03	22.99	100	1 641	3 047	2 139	6 827	24.04	44.63	31.33	100
Health Condition	No	4 895	7 711	2 902	15508	31.56	49.72	18.71	100	2 115	3 224	1 574	6 913	30.59	46.64	22.77	100
	Yes	698	1 538	1 026	3262	29.80	49.28	31.45	100	455	1 297	1 544	3 296	13.80	39.35	46.84	100
**Total**		**5 593**	**9 249**	**3 928**	**18 770**	**29.80**	**49.28**	**20.93**	**100**	**2 570**	**4 521**	**3 118**	**10 209**	**25.17**	**44.28**	**30.54**	**100**

Table S9: OR for unadjusted and adjusted models for all 3 occupational exposure groups for the binary logistic model

Industry (SIC)						
	Unadjusted			Adjusted*		
		CI95%			CI95%	
**Self-reported long-COVID**	**OR**	**Low**	**High**	**OR**	**Low**	**High**
Teaching and education	1.41	1.36	1.46	1.27	1.23	1.31
Health care	1.19	1.14	1.23	1.09	1.05	1.13
Social care	1.38	1.28	1.48	1.22	1.13	1.31
Transport (incl. storage, logistic)	1.08	1.00	1.16	1.12	1.04	1.21
Retail sector (incl. wholesale)	1.08	1.03	1.14	1.07	1.02	1.13
Hospitality (e.g. hotel, restaurant)	1.01	0.92	1.12	1.05	0.95	1.16
Food production, agriculture, farming	0.96	0.86	1.07	0.99	0.89	1.11
Personal services (e.g. hairdressers)	0.98	0.85	1.14	0.94	0.81	1.10
Information technology and communication	0.75	0.71	0.79	0.81	0.77	0.86
Financial services incl. insurance	0.78	0.74	0.82	0.81	0.77	0.85
Manufacturing or construction	0.98	0.94	1.02	1.03	0.98	1.08
Civil service or Local Government	1.12	1.06	1.17	1.06	1.01	1.11
Armed forces	0.77	0.60	0.99	0.86	0.67	1.11
Arts,Entertainment or Recreation	0.89	0.81	0.98	0.93	0.84	1.02
Other occupation sector	0.89	0.86	0.93	0.91	0.87	0.94
Not working	0.96	0.94	0.97	0.98	0.96	0.99

New occupations						
	Unadjusted			Adjusted*		
		CI95%			CI95%	
	**OR**	**Low**	**High**	**OR**	**Low**	**High**
Education	1.53	1.45	1.60	1.34	1.28	1.41
Food processing	1.16	0.97	1.38	1.10	0.92	1.31
Healthcare-office based	1.07	0.86	1.32	0.92	0.75	1.14
Healthcare-patient contact	1.14	1.08	1.20	1.04	0.98	1.10
Hospitality	1.27	1.13	1.43	1.27	1.13	1.43
Manual	0.94	0.89	1.00	1.03	0.97	1.10
Other workers-non-office based	0.93	0.88	0.98	0.98	0.93	1.05
Other workers-office based	0.90	0.88	0.92	0.90	0.88	0.92
Personal care	1.09	0.77	1.53	0.95	0.68	1.34
Police and protective services	1.27	1.15	1.40	1.31	1.18	1.45
Retail	1.10	1.02	1.20	1.08	1.00	1.17
Sanitation services	1.18	1.02	1.36	1.03	0.89	1.19
Social care	1.53	1.44	1.63	1.34	1.26	1.43
Transport-nonpublic facing	1.10	0.99	1.23	1.16	1.04	1.29
Transport-public facing	1.19	0.99	1.44	1.25	1.03	1.51
Not working	0.97	0.95	0.98	0.99	0.97	1.00

Occupation (SOC-1 digit)						
	Unadjusted			Adjusted		
		CI95%			CI95%	
	**OR**	**Low**	**High**	**OR**	**Low**	**High**
Managers, directors and senior official	0.99	0.95	1.03	0.99	0.95	1.03
Professional occupations	0.97	0.95	0.99	0.96	0.94	0.99
Associate professional and technical	0.98	0.95	1.02	1.01	0.97	1.04
Admin and secretarial	0.99	0.95	1.03	0.91	0.87	0.95
Skilled trades	0.94	0.89	1.00	1.04	0.98	1.11
Caring, leisure and other service	1.65	1.58	1.73	1.44	1.38	1.52
Sales and customer service	1.16	1.08	1.24	1.11	1.04	1.19
Process plant and machine operatives	1.07	0.99	1.16	1.14	1.05	1.23
Elementary	1.12	1.05	1.20	1.11	1.03	1.19
Not working	0.95	0.94	0.97	0.97	0.95	0.99

*Note: adjusted for age, sex, ethnicity, IMD (deprivation index), UK region of residence, Urban/Rural, Household size, past health condition

Table S10: OR for unadjusted and adjusted models for all 3 occupational exposure groups for the ordered logistic model

Industry (SIC)						
	Unadjusted			Adjusted		
		CI95%			CI95%	
**Self-reported long-COVID**	**OR**	**Low**	**High**	**OR**	**Low**	**High**
Teaching and education	0.98	0.93	1.03	0.98	0.92	1.03
Health care	1.07	1.00	1.14	1.00	0.93	1.07
Social care	1.07	0.95	1.20	0.96	0.85	1.08
Transport (incl. storage, logistic)	0.90	0.79	1.02	0.94	0.82	1.08
Retail sector (incl. wholesale)	0.89	0.81	0.98	0.91	0.83	1.00
Hospitality (e.g. hotel, restaurant)	0.76	0.64	0.90	0.82	0.68	0.98
Food production, agriculture, farming	0.99	0.82	1.20	0.98	0.80	1.19
Personal services (e.g. hairdressers)	0.95	0.73	1.23	0.94	0.71	1.23
Information technology and communication	0.69	0.63	0.77	0.79	0.71	0.88
Financial services incl. insurance	0.69	0.63	0.75	0.76	0.69	0.83
Manufacturing or construction	0.79	0.73	0.85	0.87	0.80	0.94
Civil service or Local Government	0.99	0.91	1.08	0.94	0.86	1.03
Armed forces	0.94	0.59	1.50	1.18	0.72	1.94
Arts,Entertainment or Recreation	0.73	0.62	0.87	0.81	0.68	0.97
Other occupation sector	0.81	0.76	0.87	0.85	0.80	0.91
Not working	1.26	1.23	1.30	1.21	1.17	1.26

New occupations						
	Unadjusted			Adjusted		
		CI95%			CI95%	
	**OR**	**Low**	**High**	**OR**	**Low**	**High**
Education	0.97	0.90	1.05	0.97	0.89	1.05
Food processing	0.95	0.69	1.31	0.86	0.62	1.21
Healthcare-office based	0.79	0.52	1.19	0.70	0.46	1.08
Healthcare-patient contact	1.03	0.94	1.13	0.98	0.89	1.08
Hospitality	0.89	0.72	1.09	0.91	0.74	1.12
Manual	0.91	0.82	1.02	1.01	0.90	1.13
Other workers-non-office based	0.74	0.67	0.82	0.82	0.74	0.91
Other workers-office based	0.76	0.73	0.79	0.79	0.76	0.82
Personal care	1.25	0.67	2.32	1.21	0.64	2.27
Police and protective services	0.90	0.75	1.07	0.96	0.80	1.16
Retail	1.00	0.87	1.14	1.04	0.91	1.19
Sanitation services	0.94	0.74	1.21	0.80	0.62	1.03
Social care	1.23	1.10	1.37	1.09	0.97	1.22
Transport-nonpublic facing	0.81	0.67	0.97	0.83	0.69	1.00
Transport-public facing	1.29	0.92	1.81	1.28	0.91	1.80
Not working	1.25	1.21	1.29	1.21	1.18	1.25

Occupation (SOC-1 digit)						
	Unadjusted			Adjusted		
		CI95%			CI95%	
	**OR**	**Low**	**High**	**OR**	**Low**	**High**
Managers, directors and senior official	0.77	0.71	0.83	0.82	0.75	0.88
Professional occupations	0.88	0.84	0.92	0.92	0.88	0.96
Associate professional and technical	0.81	0.77	0.86	0.87	0.82	0.92
Admin and secretarial	0.87	0.81	0.93	0.83	0.77	0.89
Skilled trades	0.83	0.75	0.92	0.93	0.84	1.04
Caring, leisure and other service	1.09	1.01	1.18	1.01	0.93	1.10
Sales and customer service	0.93	0.83	1.05	0.92	0.81	1.03
Process plant and machine operatives	0.97	0.85	1.12	1.01	0.87	1.16
Elementary	0.96	0.84	1.08	0.92	0.81	1.04
Not working	1.27	1.23	1.31	1.22	1.18	1.26

*Note: adjusted for age, sex, ethnicity, IMD (deprivation index), UK region of residence, Urban/Rural, Household size, past health condition

Table S11a: Probabilities (marginal means) and 95% confidence intervals (95% CI) of self-reported long-COVID by industry (SIC), occupations and occupational SOC (1-digit) groups

		Unadjusted			Adjusted		
			95%CI			95%CI	
		Prob	Low	High	Prob	Low	High
Industry (SIC)	Teaching and education	0.127	0.123	0.130	0.191	0.183	0.200
	Health care	0.109	0.105	0.113	0.159	0.150	0.168
	Social care	0.124	0.116	0.132	0.182	0.164	0.200
	Transport (incl. storage, logistic)	0.100	0.093	0.106	0.188	0.170	0.205
	Retail sector (incl. wholesale)	0.100	0.095	0.105	0.179	0.166	0.192
	Hospitality (e.g. hotel, restaurant)	0.095	0.086	0.103	0.165	0.142	0.187
	Food production, agriculture, farming	0.090	0.081	0.099	0.159	0.136	0.183
	Personal services (e.g. hairdressers)	0.092	0.079	0.104	0.145	0.115	0.176
	IT and communication	0.071	0.068	0.075	0.143	0.131	0.154
	Financial services incl. insurance	0.074	0.071	0.078	0.139	0.129	0.150
	Manufacturing or construction	0.091	0.088	0.095	0.167	0.157	0.177
	Civil service or Local Government	0.103	0.099	0.108	0.183	0.171	0.195
	Armed forces	0.074	0.057	0.091	0.139	0.089	0.189
	Arts, Entertainment or Recreation	0.084	0.076	0.092	0.152	0.130	0.174
	Other occupation sector	0.084	0.081	0.087	0.157	0.149	0.165
	Not working	0.090	0.088	0.091	0.172	0.167	0.177
Occupations	education	0.135	0.129	0.140	0.194	0.182	0.206
	food processing	0.106	0.089	0.122	0.171	0.130	0.211
	healthcare-office based	0.098	0.079	0.117	0.154	0.109	0.198
	healthcare-patient contact	0.104	0.099	0.109	0.146	0.134	0.158
	hospitality	0.115	0.103	0.127	0.188	0.157	0.218
	manual	0.088	0.083	0.093	0.168	0.154	0.182
	other workers-non-office based	0.086	0.082	0.091	0.167	0.154	0.181
	other workers-office based	0.084	0.082	0.086	0.153	0.147	0.158
	personal care	0.100	0.069	0.130	0.147	0.075	0.219
	police and protective services	0.114	0.104	0.125	0.206	0.180	0.232
	retail	0.101	0.094	0.108	0.181	0.162	0.201
	sanitation services	0.107	0.093	0.121	0.154	0.123	0.185
	social care	0.135	0.128	0.142	0.189	0.173	0.206
	transport-nonpublic facing	0.101	0.091	0.111	0.205	0.178	0.232
	transport-public facing	0.108	0.090	0.127	0.202	0.153	0.251
	Not working	0.090	0.088	0.091	0.172	0.167	0.177
Occupations (SOC)	Managers, directors & senior officials	0.093	0.089	0.096	0.159	0.149	0.169
	Professional occupations	0.091	0.089	0.093	0.156	0.150	0.162
	Associate professional & technical	0.092	0.089	0.096	0.167	0.159	0.175
	Admin and secretarial	0.093	0.090	0.097	0.160	0.150	0.169
	Skilled trades	0.089	0.084	0.094	0.170	0.156	0.184
	Caring, leisure and other service	0.146	0.140	0.152	0.204	0.191	0.216
	Sales and customer service	0.107	0.100	0.113	0.200	0.183	0.217
	Process plant & machine operatives	0.100	0.093	0.107	0.178	0.159	0.197
	Elementary	0.104	0.097	0.111	0.184	0.167	0.201
	Not working	0.090	0.088	0.091	0.172	0.167	0.178

Table 11b: Probabilities (marginal means) and 95% confidence intervals (95% CI) of reduced function by industry (SIC), occupations and occupational SOC (1-digit) groups

		Unadjusted									Adjusted								
		Not at all			Yes, a little			Yes, a lot			Not at all			Yes, a little			Yes, a lot		
			95%			95%			95%			95%			95%			95%	
		Prob	Low	High	Prob	Low	High	Prob	Low	High	Prob	Low	High	Prob	Low	High	Prob	Low	High
Industry (SIC)	Teaching and education	0.282	0.271	0.294	0.480	0.474	0.486	0.238	0.227	0.248	0.283	0.270	0.295	0.480	0.474	0.485	0.238	0.227	0.249
	Health care	0.265	0.251	0.279	0.481	0.475	0.487	0.254	0.240	0.267	0.278	0.263	0.292	0.480	0.474	0.486	0.242	0.230	0.255
	Social care	0.265	0.241	0.289	0.481	0.475	0.487	0.254	0.231	0.277	0.286	0.262	0.311	0.479	0.473	0.485	0.235	0.213	0.256
	Transport (incl. storage, logistic)	0.300	0.272	0.328	0.477	0.470	0.485	0.222	0.200	0.245	0.289	0.263	0.316	0.479	0.472	0.485	0.232	0.209	0.255
	Retail sector (incl. wholesale)	0.302	0.282	0.323	0.477	0.470	0.484	0.221	0.204	0.237	0.296	0.276	0.316	0.478	0.471	0.484	0.226	0.209	0.243
	Hospitality (e.g. hotel, restaurant)	0.337	0.298	0.376	0.469	0.456	0.482	0.194	0.167	0.222	0.318	0.280	0.356	0.473	0.463	0.484	0.208	0.180	0.237
	Food production,agriculture,farming	0.280	0.240	0.319	0.480	0.473	0.487	0.240	0.204	0.276	0.282	0.243	0.321	0.480	0.473	0.487	0.238	0.203	0.273
	Personal services (e.g. hairdressers)	0.289	0.235	0.344	0.479	0.470	0.488	0.232	0.184	0.279	0.290	0.236	0.345	0.479	0.469	0.488	0.231	0.184	0.278
	IT and communication	0.357	0.333	0.380	0.462	0.452	0.472	0.181	0.166	0.196	0.325	0.303	0.348	0.472	0.464	0.479	0.203	0.186	0.220
	Financial services incl. insurance	0.359	0.338	0.380	0.461	0.452	0.471	0.180	0.166	0.194	0.334	0.313	0.354	0.469	0.462	0.477	0.197	0.182	0.212
	Manufacturing or construction	0.329	0.312	0.346	0.471	0.464	0.478	0.200	0.187	0.213	0.306	0.289	0.323	0.476	0.470	0.482	0.218	0.204	0.232
	Civil service or Local Government	0.280	0.263	0.298	0.480	0.474	0.486	0.240	0.224	0.256	0.290	0.272	0.308	0.479	0.473	0.485	0.232	0.216	0.247
	Armed forces	0.291	0.194	0.389	0.479	0.464	0.494	0.230	0.146	0.313	0.247	0.158	0.336	0.480	0.473	0.488	0.273	0.179	0.367
	Arts,Entertainment or Recreation	0.344	0.304	0.384	0.466	0.453	0.480	0.190	0.162	0.217	0.320	0.282	0.357	0.473	0.462	0.484	0.207	0.179	0.236
	Other occupation sector	0.322	0.307	0.337	0.473	0.466	0.479	0.205	0.194	0.217	0.309	0.295	0.324	0.475	0.469	0.482	0.216	0.204	0.227
	Not working	0.234	0.226	0.241	0.479	0.474	0.485	0.287	0.279	0.295	0.242	0.234	0.250	0.480	0.479	0.497	0.278	0.270	0.286
Occupations	education	0.283	0.266	0.299	0.478	0.471	0.484	0.240	0.225	0.254	0.282	0.266	0.298	0.477	0.471	0.484	0.241	0.226	0.256
	food processing	0.287	0.221	0.353	0.477	0.467	0.487	0.236	0.177	0.294	0.304	0.236	0.373	0.474	0.460	0.488	0.221	0.170	0.285
	healthcare-office based	0.326	0.235	0.417	0.470	0.445	0.494	0.204	0.137	0.272	0.347	0.255	0.440	0.463	0.434	0.493	0.189	0.113	0.237
	healthcare-patient contact	0.270	0.251	0.290	0.478	0.472	0.485	0.251	0.233	0.269	0.280	0.261	0.300	0.477	0.471	0.484	0.242	0.222	0.258
	hospitality	0.301	0.258	0.344	0.475	0.466	0.485	0.224	0.189	0.260	0.294	0.253	0.335	0.476	0.467	0.484	0.230	0.199	0.272
	manual	0.295	0.272	0.317	0.476	0.469	0.483	0.229	0.210	0.249	0.273	0.252	0.295	0.478	0.472	0.484	0.249	0.235	0.278
	other workers-non-office based	0.340	0.317	0.363	0.466	0.457	0.475	0.194	0.178	0.211	0.316	0.294	0.338	0.472	0.464	0.479	0.212	0.196	0.232
	other workers-office based	0.335	0.325	0.345	0.467	0.461	0.474	0.198	0.190	0.206	0.323	0.313	0.333	0.470	0.464	0.476	0.207	0.199	0.216
	personal care	0.234	0.123	0.346	0.477	0.460	0.494	0.289	0.161	0.417	0.241	0.130	0.352	0.478	0.465	0.490	0.281	0.152	0.396
	police and protective services	0.298	0.261	0.336	0.476	0.467	0.484	0.226	0.195	0.258	0.284	0.247	0.320	0.477	0.470	0.484	0.239	0.207	0.274
	retail	0.277	0.249	0.304	0.478	0.472	0.485	0.245	0.220	0.271	0.268	0.242	0.294	0.478	0.472	0.484	0.254	0.227	0.279
	sanitation services	0.288	0.237	0.339	0.477	0.468	0.486	0.235	0.190	0.280	0.320	0.267	0.374	0.471	0.457	0.485	0.209	0.164	0.247
	social care	0.237	0.216	0.257	0.477	0.471	0.484	0.286	0.263	0.309	0.260	0.239	0.281	0.478	0.472	0.485	0.262	0.239	0.282
	transport-nonpublic facing	0.320	0.280	0.361	0.471	0.459	0.482	0.209	0.178	0.240	0.313	0.273	0.352	0.472	0.462	0.483	0.215	0.185	0.248
	transport-public facing	0.228	0.168	0.288	0.476	0.463	0.488	0.296	0.225	0.367	0.231	0.172	0.289	0.476	0.465	0.487	0.293	0.229	0.369
	Not working	0.234	0.227	0.242	0.477	0.471	0.483	0.289	0.281	0.297	0.240	0.232	0.248	0.477	0.471	0.484	0.282	0.272	0.290
Occupations (SOC)	Managers, directors & senior officials	0.336	0.319	0.354	0.467	0.460	0.475	0.196	0.184	0.209	0.320	0.303	0.337	0.472	0.465	0.478	0.209	0.195	0.222
	Professional occupations	0.306	0.296	0.317	0.475	0.469	0.481	0.219	0.210	0.227	0.296	0.286	0.306	0.476	0.470	0.482	0.228	0.219	0.237
	Associate professional & technical	0.323	0.309	0.337	0.471	0.465	0.478	0.206	0.196	0.217	0.307	0.294	0.321	0.474	0.468	0.480	0.219	0.207	0.230
	Admin and secretarial	0.308	0.292	0.324	0.474	0.468	0.481	0.217	0.204	0.230	0.317	0.301	0.333	0.472	0.465	0.479	0.211	0.198	0.223
	Skilled trades	0.318	0.296	0.341	0.472	0.464	0.480	0.209	0.192	0.227	0.293	0.271	0.315	0.477	0.470	0.483	0.230	0.212	0.249
	Caring, leisure and other service	0.262	0.246	0.278	0.479	0.474	0.485	0.259	0.243	0.275	0.277	0.260	0.294	0.478	0.473	0.484	0.244	0.229	0.260
	Sales and customer service	0.294	0.269	0.319	0.477	0.470	0.484	0.229	0.207	0.250	0.296	0.271	0.320	0.476	0.469	0.483	0.228	0.207	0.249
	Process plant & machine operatives	0.285	0.256	0.314	0.478	0.471	0.485	0.237	0.211	0.263	0.278	0.249	0.306	0.478	0.472	0.485	0.244	0.218	0.270
	Elementary	0.288	0.262	0.314	0.478	0.471	0.484	0.234	0.211	0.257	0.295	0.270	0.321	0.476	0.469	0.483	0.228	0.206	0.250
	Not working	0.234	0.226	0.241	0.478	0.472	0.483	0.289	0.280	0.297	0.242	0.234	0.250	0.478	0.473	0.484	0.280	0.271	0.288

Table S12: Comparison of odds ratios between one observation person and Panel analyses (Industrial SIC groups)

Industries	Long Covid (adjusted model)						Reduced function (adjusted model)					
	One observation per person			Panel			One observation per person			Panel		
	OR	95% CI		OR	95% CI		OR	95% CI		OR	95% CI	
		Lower	Higher		Lower	Higher		Lower	Higher		Lower	Higher
**Teaching and education**	1.27	1.23	1.31	1.39	1.31	1.49	0.98	0.92	1.03	0.92	0.85	0.98
**Health care**	1.09	1.05	1.13	1.25	1.17	1.35	1.00	0.93	1.07	0.85	0.79	0.92
**Social care**	1.22	1.13	1.31	1.28	1.12	1.45	0.96	0.85	1.08	0.82	0.71	0.94
**Transport (incl. storage, logistic)**	1.12	1.04	1.21	1.20	1.03	1.40	0.94	0.82	1.08	0.91	0.78	1.07
**Retail sector (incl. wholesale)**	1.07	1.02	1.13	1.05	0.95	1.16	0.91	0.83	1.00	0.87	0.78	0.97
**Hospitality (e.g. hotel, restaurant)**	1.05	0.95	1.16	1.15	1.00	1.32	0.82	0.68	0.98	0.89	0.75	1.06
**Food production, agriculture, farming**	0.99	0.89	1.11	0.94	0.73	1.21	0.98	0.80	1.19	1.03	0.83	1.29
**Personal services (e.g. hairdressers)**	0.94	0.81	1.10	0.93	0.74	1.16	0.94	0.71	1.23	0.88	0.68	1.14
**Information technology and communication**	0.81	0.77	0.86	0.69	0.59	0.81	0.79	0.71	0.88	0.70	0.62	0.80
**Financial services incl. insurance**	0.81	0.77	0.85	0.68	0.59	0.79	0.76	0.69	0.83	0.68	0.60	0.76
**Manufacturing or construction**	1.03	0.98	1.08	1.08	0.98	1.20	0.87	0.80	0.94	0.82	0.74	0.90
**Civil service or Local Government**	1.06	1.01	1.11	1.10	1.00	1.21	0.94	0.86	1.03	0.84	0.76	0.93
**Armed forces**	0.86	0.67	1.11	0.73	0.35	1.52	1.18	0.72	1.94	1.38	0.73	2.63
**Arts,Entertainment or Recreation**	0.93	0.84	1.02	0.90	0.73	1.10	0.81	0.68	0.97	0.85	0.69	1.04
**Other occupation sector**	0.91	0.87	0.94	0.89	0.82	0.96	0.85	0.80	0.91	0.75	0.70	0.82
**Not working**	0.98	0.96	0.99	0.95	0.92	0.98	1.21	1.17	1.26	1.51	1.44	1.57

*Note: adjusted for age, sex, ethnicity, IMD (deprivation index), UK region of residence, Urban/Rural, Household size, past health condition

Table S13: Comparison of odds ratios between one observation person and Panel analyses (Occupational groups)

Occupations	Long Covid (adjusted model)						Reduced function (adjusted model)					
	One observation per person			Panel			One observation per person			Panel		
	OR	95% CI		OR	95% CI		OR	95% CI		OR	95% CI	
		Lower	Higher		Lower	Higher		Lower	Higher		Lower	Higher
**Education**	1.34	1.28	1.41	1.58	1.45	1.72	0.97	0.89	1.05	0.90	0.82	1.00
**food processing**	1.10	0.92	1.31	0.99	0.75	1.32	0.86	0.62	1.21	0.82	0.58	1.16
**Healthcare-office based**	0.92	0.75	1.14	0.80	0.52	1.25	0.70	0.46	1.08	0.61	0.37	1.00
**Healthcare-patient contact**	1.04	0.98	1.10	1.17	1.05	1.30	0.98	0.89	1.08	0.87	0.78	0.98
**Hospitality**	1.27	1.13	1.43	1.37	1.16	1.63	0.91	0.74	1.12	0.94	0.76	1.16
**Manual**	1.03	0.97	1.10	1.08	0.94	1.24	1.01	0.90	1.13	0.94	0.83	1.08
**Other workers-non-office based**	0.98	0.93	1.05	0.99	0.87	1.13	0.82	0.74	0.91	0.81	0.72	0.93
**Other workers-office based**	0.90	0.88	0.92	0.86	0.82	0.90	0.79	0.76	0.82	0.69	0.66	0.72
**Personal care**	0.95	0.68	1.34	1.16	0.85	1.60	1.21	0.64	2.27	0.77	0.53	1.13
**Police and protective services**	1.31	1.18	1.45	1.69	1.38	2.07	0.96	0.80	1.16	0.83	0.67	1.03
**Retail**	1.08	1.00	1.17	1.18	1.02	1.35	1.04	0.91	1.19	0.96	0.83	1.11
**Sanitation services**	1.03	0.89	1.19	0.98	0.76	1.27	0.80	0.62	1.03	0.70	0.53	0.93
**Social care**	1.34	1.26	1.43	1.44	1.29	1.62	1.09	0.97	1.22	0.83	0.73	0.94
**Transport-nonpublic facing**	1.16	1.04	1.29	1.12	0.88	1.43	0.83	0.69	1.00	0.67	0.53	0.85
**Transport-public facing**	1.25	1.03	1.51	1.43	1.04	1.95	1.28	0.91	1.80	0.91	0.65	1.27
**Not working**	0.99	0.97	1.00	0.95	0.92	0.98	1.21	1.18	1.25	1.52	1.45	1.58

*Note: adjusted for age, sex, ethnicity, IMD (deprivation index), UK region of residence, Urban/Rural, Household size, past health condition

Table S14: Comparison of odds ratios between one observation person and Panel analyses (1-digit SOC groups)

**1-digit SOC**	**Long Covid (adjusted model)**						**Reduced function (adjusted model)**					
	**One observation per person**			**Panel**			**One observation per person**			**Panel**		
	**OR**	**95% CI**		**OR**	**95% CI**		**OR**	**95% CI**		**OR**	**95% CI**	
		**Lower**	**Higher**		**Lower**	**Higher**		**Lower**	**Higher**		**Lower**	**Higher**
**Managers, directors and senior official**	0.99	0.95	1.03	0.94	0.90	1.09	0.82	0.75	0.88	0.73	0.66	0.81
**Professional occupations**	0.96	0.94	0.99	1.03	0.89	0.99	0.92	0.88	0.96	0.84	0.80	0.89
**Associate professional and technical**	1.01	0.97	1.04	0.88	0.96	1.11	0.87	0.82	0.92	0.78	0.72	0.84
**Admin and secretarial**	0.91	0.87	0.95	1.15	0.81	0.95	0.83	0.77	0.89	0.71	0.65	0.77
**Skilled trades**	1.04	0.98	1.11	1.56	1.01	1.31	0.93	0.84	1.04	0.92	0.81	1.05
**Caring, leisure and other service**	1.44	1.38	1.52	1.11	1.44	1.69	1.01	0.93	1.10	0.86	0.78	0.94
**Sales and customer service**	1.11	1.04	1.19	1.30	0.99	1.24	0.92	0.81	1.03	0.91	0.80	1.05
**Process plant and machine operatives**	1.14	1.05	1.23	1.09	1.11	1.52	1.01	0.87	1.16	0.85	0.72	1.01
**Elementary**	1.11	1.03	1.19	0.95	0.97	1.23	0.92	0.81	1.04	0.89	0.77	1.02
**Not working**	0.97	0.95	0.99	0.94	0.91	0.98	1.22	1.18	1.26	1.52	1.46	1.59

*Note: adjusted for age, sex, ethnicity, IMD (deprivation index), UK region of residence, Urban/Rural, Household size, past health condition

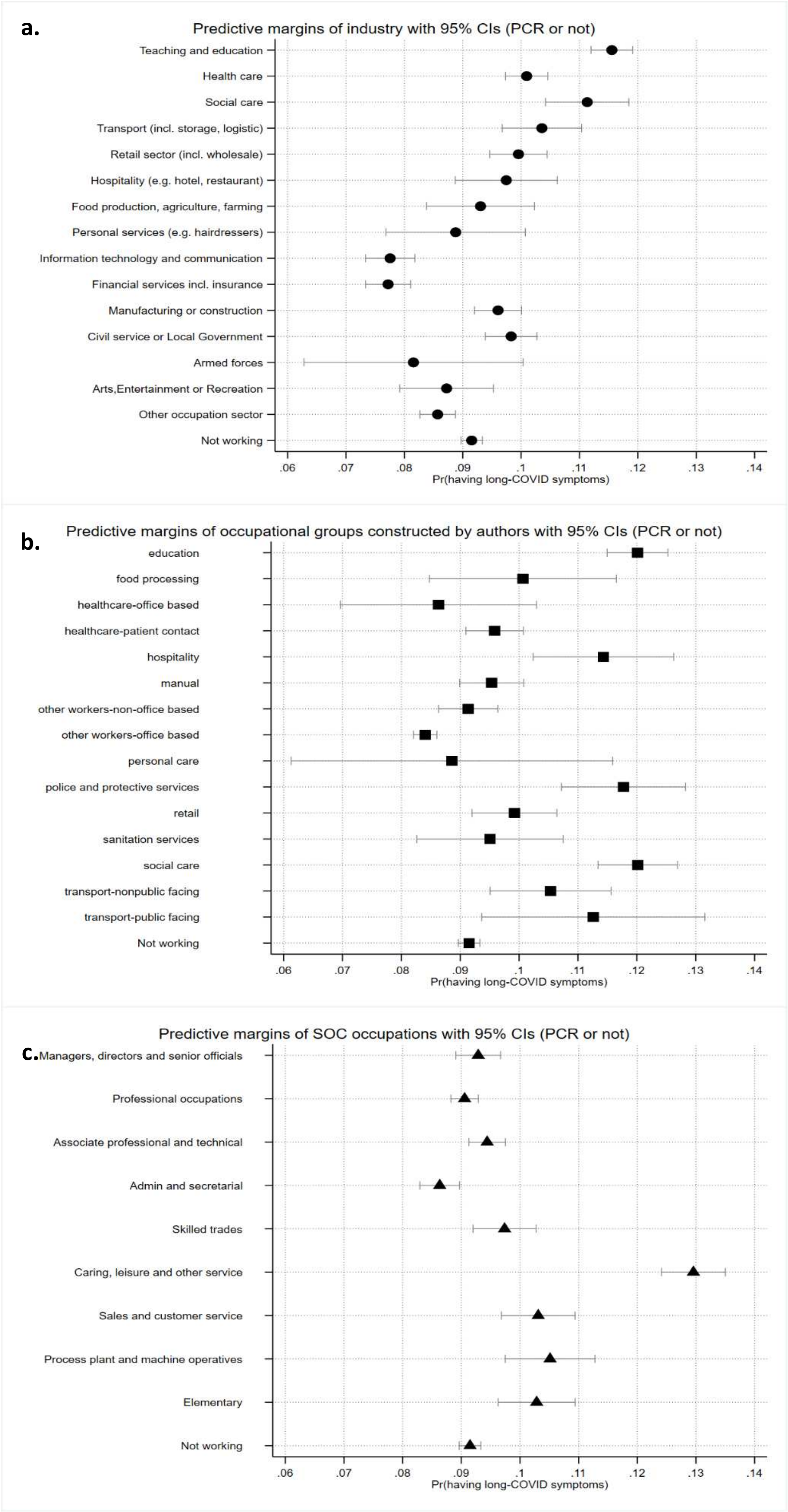

Figure S1: Predictive margins of long-COVID by (a) industry; (b) occupational groups; and (c) major SOC groups

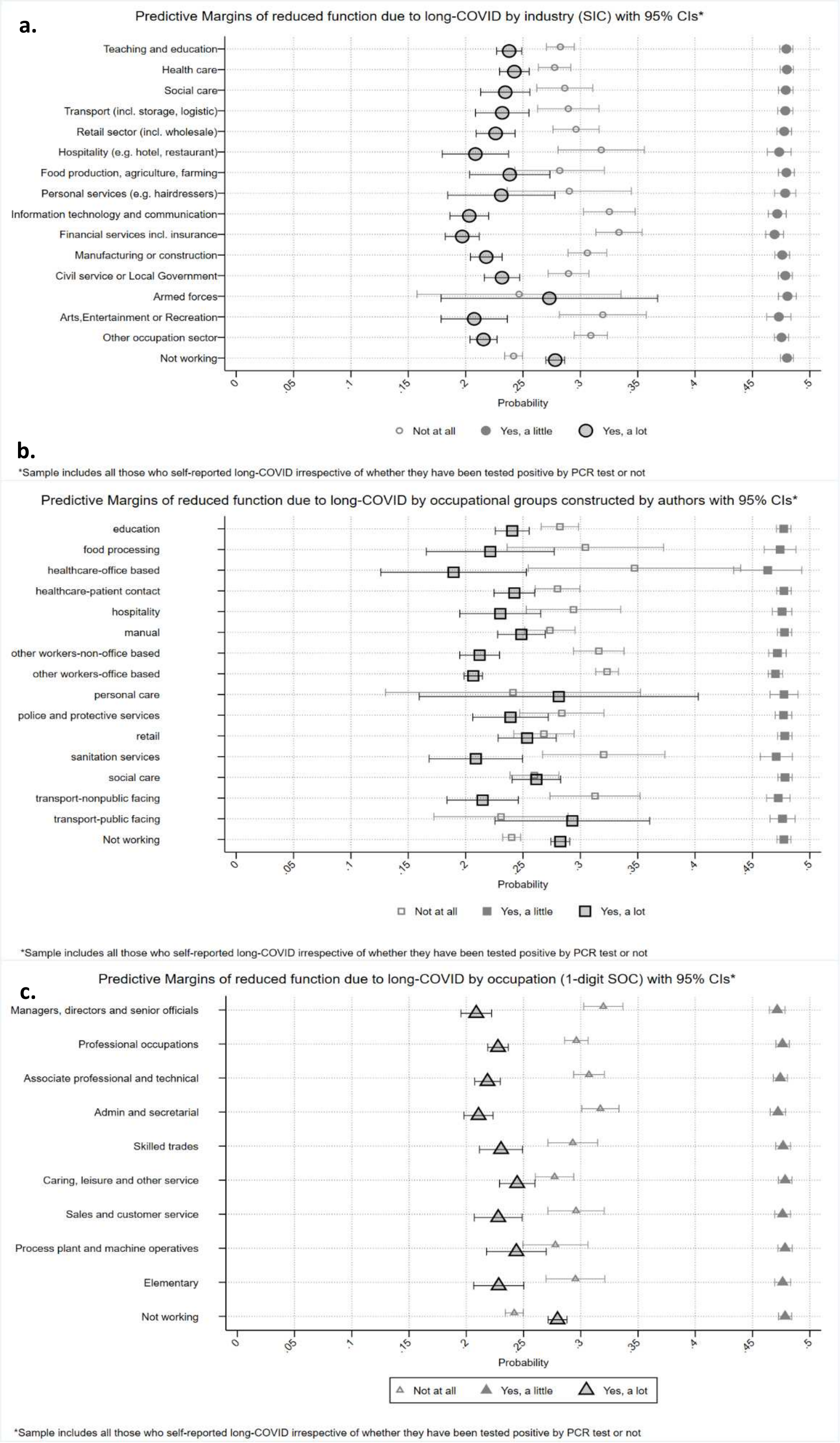

Figure S2: Predictive margins of reduced activities due to long-COVID by (a) industry; (b) occupational groups; and (c) major SOC groups

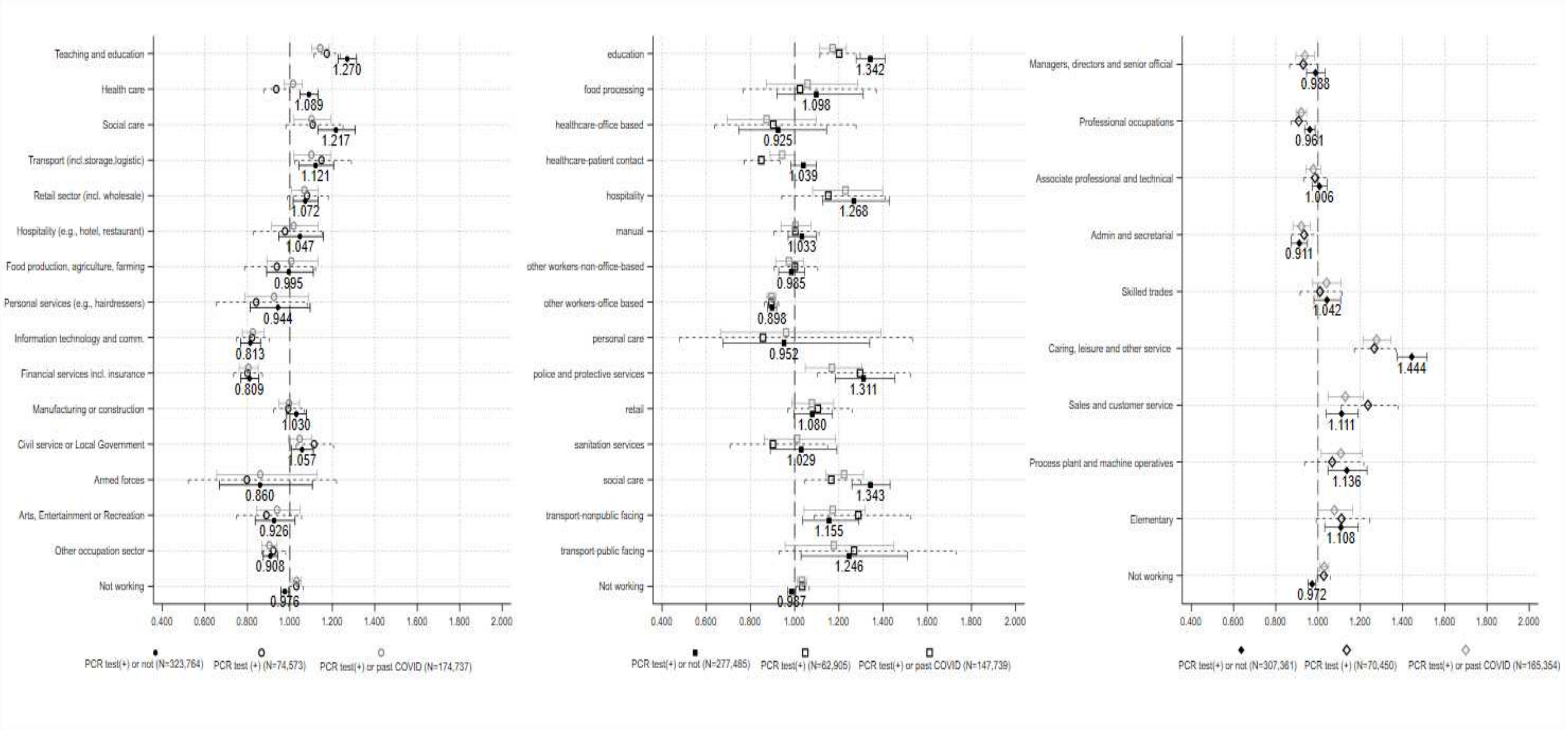

Figure S3: OR comparison between all three samples across all exposure groups (i: PCR test(+) or not, ii: PCR test (+) and iii) PCR test (+) or past COVID) (Long-COVID)

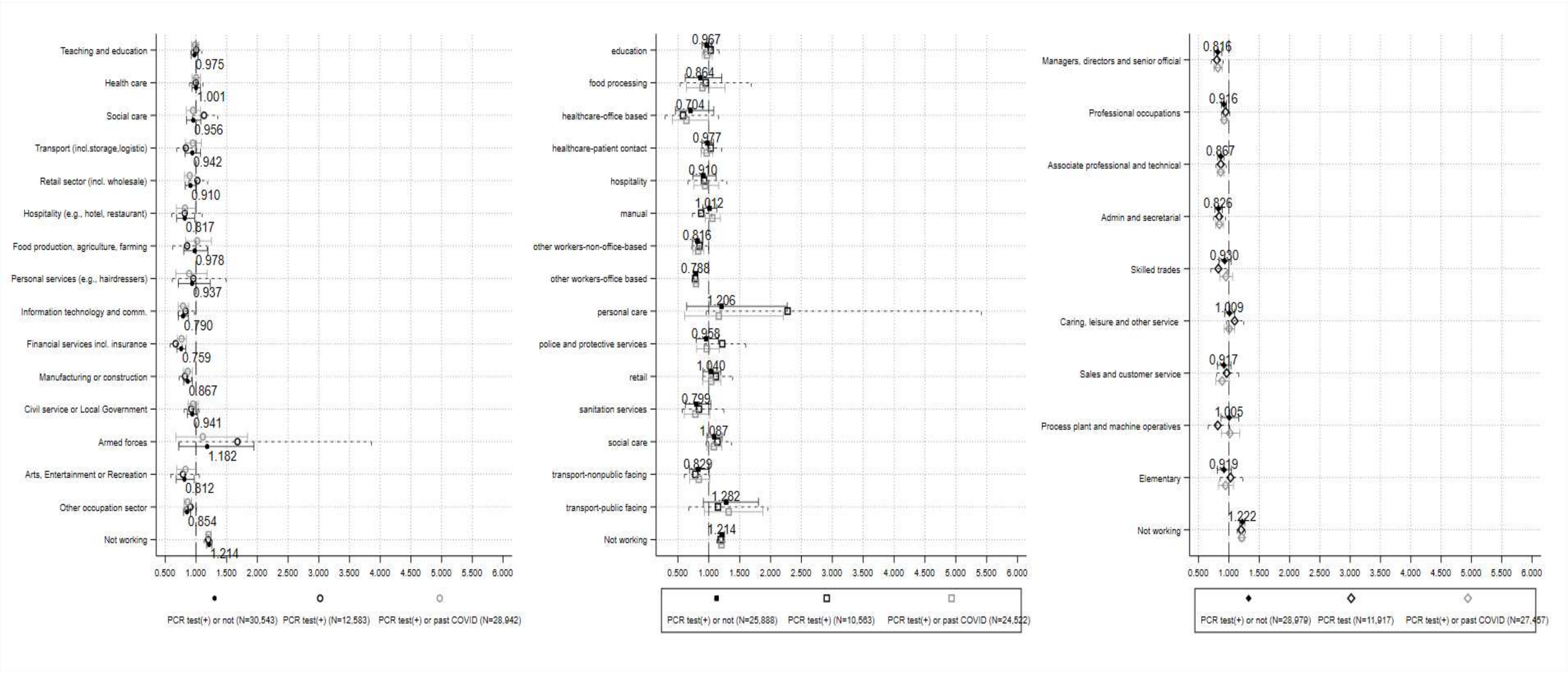

FigureS4: OR comparison between all three samples across all exposure groups (i: PCR test(+) or not, ii: PCR test (+) and iii) PCR test (+) or past COVID) (Reduced function)

1 Oude Hengel KM, Burdorf A, Pronk A et al. Exposure to a SARS-CoV-2 infection at work: development of an international job exposure matrix (COVID-19-JEM). *Scand J Work Environ Health* 2022;48:61-70.
